# Synthetic C_1_ metabolism in *Pseudomonas putida* enables strict formatotrophy and methylotrophy via the reductive glycine pathway

**DOI:** 10.1128/mbio.01976-25

**Published:** 2025-08-18

**Authors:** Justine Turlin, Maria V. G. Alván-Vargas, Òscar Puiggené, Stefano Donati, Sebastian Wenk, Pablo I. Nikel

**Affiliations:** 1 The Novo Nordisk Foundation Center for Biosustainability, Technical University of Denmark 5205, Lyngby, Denmark; 2 Max Planck Institute of Molecular Plant Physiology 28322, Potsdam, Germany; 3 Faculty of Science and Engineering, University of Groningen 3647, Groningen, the Netherlands; The University of Oklahoma, Norman, Oklahoma, USA

**Keywords:** metabolic engineering, synthetic biology, synthetic metabolism, *Pseudomonas*, growth coupling, formate, methanol

## Abstract

Formate and methanol are promising alternatives to sugar-based feedstocks for biotechnological applications. These one-carbon (C_1_) substrates can be sustainably produced from CO_2_ and renewable electricity and assimilated by both native and engineered microbial systems. However, their broader adoption is limited by the narrow range of bacterial hosts capable of efficient methanol and formate utilization. In this study, the industrially relevant soil bacterium *Pseudomonas putida* was metabolically engineered to assimilate formate and methanol as sole carbon and energy sources via the linear reductive glycine pathway. Initial strains were optimized for formate assimilation using acetate for energy conservation through adaptive laboratory evolution (ALE), leading to a substantial reduction in doubling time under mixotrophic conditions. Key mutations emerged in the promoter regions of synthetic pathway genes and within the native genome. Strictly formatotrophic growth, with a doubling time of ca. 28 h, was achieved by integrating a formate dehydrogenase gene either on a plasmid or chromosomally as a mini-Tn*5* module, combined with growth-coupled selection. The resulting strain, *P. putida* rG·F, was then re-engineered by replacing the formate dehydrogenase with an engineered methanol dehydrogenase from *Cupriavidus necator*. Following ALE, an isolate displaying full methylotrophy, *P. putida* rG·M, grew on methanol with a doubling time of ca. 24 h. These efforts demonstrate the feasibility of constructing robust C_1_-assimilating *P. putida* strains and highlight the substrate versatility of this bacterium for bioproduction. Integrating evolutionary engineering with synthetic biology tools has expanded the range of viable microbial hosts for efficient C_1_ feedstock utilization.

**IMPORTANCE:**

Soluble C_1_ feedstocks, such as formate and methanol, have gained attention as sustainable substrates for biotechnology, with the potential to reduce greenhouse gas emissions and reliance on sugar-based resources. Despite their promise, the metabolic assimilation of these compounds remains uncharacterized in robust bacterial hosts beyond a few model species. *Pseudomonas putida*, known for its metabolic versatility and industrial relevance, has lacked the ability to grow solely on C_1_ compounds. This study is a first-case example of strict synthetic formatotrophy and methylotrophy in any *Pseudomonas* species, enabling growth on formate and methanol as sole carbon and energy sources. Through pathway rewiring and adaptive laboratory evolution, key metabolic and regulatory adaptations were identified that enabled efficient C_1_ assimilation. These findings not only expand the known capabilities of *P. putida* but also open directions for its deployment in carbon-efficient biomanufacturing. This study sets a precedent for leveraging non-model microorganisms in the development of scalable, carbon-efficient bioprocesses.

## INTRODUCTION

Since the Industrial Revolution, CO_2_ emissions have increased with the expansion of industries and population. The accumulated atmospheric CO_2_ traps radiation from Earth’s surface, causing global warming (1). Achieving zero-CO_2_ emissions by 2050 requires intense efforts in reducing emissions and enhancing CO_2_ capture. Another key objective is to convert captured CO_2_ into value-added products (e.g., fuels, chemicals, and materials) through chemical, photochemical, electrochemical, or biological pathways—or their combinations (2, 3). Chemical conversion has a limited product spectrum and demands harsh conditions with low specificity and high energy consumption (4, 5), whereas biological systems, operating under mild conditions, offer a more sustainable option for generating value-added chemicals (6, 7). Gas fermentation is an established process in which CO_2_ or CO is converted by acetogens through the anaerobic Wood-Ljungdahl pathway (8). However, natural acetogens only yield a limited range of useful products, e.g., ethanol and butanol (9). Chemolithotrophs use external inorganic electron donors to fix CO_2_ (10), but direct electron transfer from electrodes remains restricted by low current density and large electrode areas, posing challenges for industrial-scale adoption. Several factors guide the selection of CO_2_-derived feedstocks, i.e., oxidation state, thermodynamic feasibility, and compatibility with co-substrates (11). Unlike gaseous substrates, soluble C_1_ mediators—e.g., formate and methanol, which can be obtained by photo- or electrochemical CO_2_ reduction (12, 13)—overcome mass transfer issues. Formate is attractive due to its high water solubility and limited toxicity (14). Methanol, more reduced than formate, requires fewer reducing equivalents for assimilation (15), influencing NAD(P)H availability for microbial growth and bioproduction (16). However, relatively few bacteria have been engineered for efficient assimilation of formate and methanol toward producing value-added products (17, 18).

The reductive glycine pathway (rGlyP) is an efficient aerobic route for formate assimilation (19–21) that has been successfully implemented in a few industrially relevant bacteria, including *Escherichia coli* (22–24) and *Cupriavidus necator* (25). A synthetic *E. coli* formatotroph was further engineered to produce lactate (26), showcasing the possibility of establishing a formate-based bioeconomy (27). The modular nature of the rGlyP has also been harnessed for enabling synthetic methylotrophy in *E. coli* (22). However, the narrow set of organisms subjected to this engineering approach constrains widespread adoption of synthetic formatotrophy and methylotrophy for industrial applications. Robust microbial hosts for engineering synthetic C_1_ assimilation should be selected based on their inherent properties, e.g., tolerance to substrates and intermediates (28). Aerobic strains are especially relevant since using O_2_ as the terminal electron acceptor broadens the range of possible products (29).

The soil bacterium *Pseudomonas putida* stands out as a robust platform for bioproduction owing to its innate resistance to toxic compounds and stress (30–34). Its natural formate tolerance (35) and native dehydrogenases for methanol oxidation (36) make it an excellent candidate for establishing synthetic C_1_ assimilation. We recently engineered *P. putida* with the rGlyP to assimilate formate under mixotrophic conditions, using acetate for energy conservation (35). Building on these efforts, in this study, we combined rational engineering, pathway modularization, growth-coupled selection, and adaptive laboratory evolution (ALE) to establish strict formatotrophic and methylotrophic growth in engineered *P. putida* strains. Quantitative physiology experiments, ^13^C-tracer labeling, and reverse engineering of key mutations acquired during ALE validated the C_1_-trophic phenotypes. These efforts expand the range of synthetic bacteria that can assimilate formate and methanol toward bolstering a robust C_1_ bioeconomy.

## RESULTS

### Engineering strategy to establish synthetic formatotrophy and methylotrophy in *P*. *putida*

**Fig 1:**
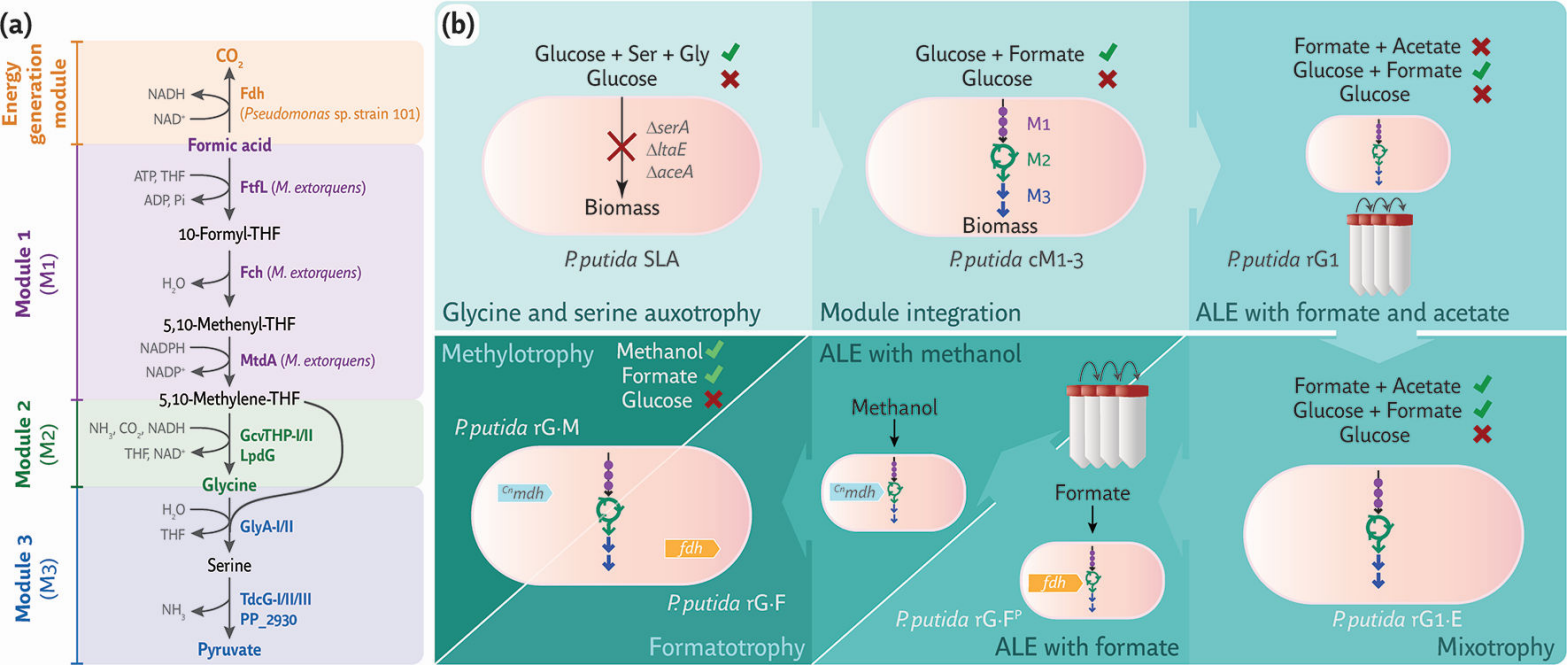
Workflow for establishing synthetic formatotrophy and methylotrophy in *P. putida*. (**a**) The reductive glycine pathway (rGlyP, represented here in its formatotrophic architecture) is divided into three modules, composed of both endogenous and heterologous (*Methylobacterium extorquens* and *Pseudomonas* sp. strain 101) activities. In *P. putida* KT2440, two sets of genes encode GlyA (serine hydroxymethyl transferase) and GcvTHP (the glycine cleavage system, GCS), while three separate genes are connected to the TdcG activity (L-serine deaminase). *Fdh*, formate dehydrogenase; *FtfL*, formate-THF ligase; *Fch*, 5,10-methenyl-THF cyclohydrolase; *MtdA*, 5,10-methylene-THF dehydrogenase; *GcvTHP*, GCS proteins T, H, and P; *LpdG*, dihydrolipoyl dehydrogenase; and *TFH*, tetrahydrofolate. (**b**) The engineering strategy employed in this study combined rational engineering with adaptive laboratory evolution. Microbial growth on specific substrate combinations, including glycine (Gly) and serine (Ser), was evaluated throughout the workflow. The final engineered strains, termed rG·F and rG·M, represent a synthetic *P. putida* formatotroph and methylotroph, respectively. *^Cn^mdh*, NAD^+^-dependent methanol dehydrogenase from *Cupriavidus necator*.

The canonical rGlyP spans three metabolic modules (Fig. 1a). The first module, termed M1, encodes three enzymes from *Methylobacterium extorquens*—formate-tetrahydrofolate (THF) ligase (FtfL), methenyl-THF cyclohydrolase (Fch), and methylene-THF dehydrogenase (MtdA)—that convert formate into 5,10-methylene-THF (22). The second module, M2, is the native glycine cleavage system (GCS) of the selected host, which condenses 5,10-methylene-THF, NH_3_, and CO_2_ to form the C_2_ amino acid glycine. In this configuration, the GCS operates in the opposite direction of its native flow, requiring a CO_2_-enriched atmosphere to drive the condensation reaction forward. The third module, M3, comprises the endogenous genes encoding L-serine hydroxymethyltransferase (GlyA) and L-serine deaminase (TdcG), condensing glycine with another 5,10-methylene-THF molecule to produce the C_3_ amino acid serine, subsequently deaminated into pyruvate. Pyruvate serves as the entry point into the host’s central carbon metabolism. An additional energy generation module (EGM) supplies NADH—thus supporting energy conservation—through a formate dehydrogenase (Fdh) that oxidizes formate into CO_2_. Previous efforts to establish synthetic formatotrophy in *E. coli* adopted the Fdh from *Pseudomonas* sp. strain 101 as the key activity within the EGM (22).

By adopting this modular, stepwise approach combined with growth-coupled selection (37), we previously established synthetic C_1_ assimilation in *P. putida* (35), where formate served as the main carbon source and acetate was processed through the tricarboxylic acid (TCA) cycle to supply catabolic reducing power—ultimately conserving energy. Building on that strategy, we sought to achieve full formatotrophy and methylotrophy, enabling formate or methanol to supply all carbon required for biomass and energy conservation. The strains resulting from each engineering step—where forward design was combined with ALE under formatotrophic or methylotrophic conditions—were systematically evaluated in minimal salt medium (MSM) containing different carbon source combinations that allowed for direct selection of active metabolic modules (Fig. 1b). The individual engineering steps and their outcomes are detailed in the sections below.

### A stepwise, rational, and evolutionary engineering approach improves formate-dependent growth of a mixotrophic *P*. *putida* strain

**Fig 2:**
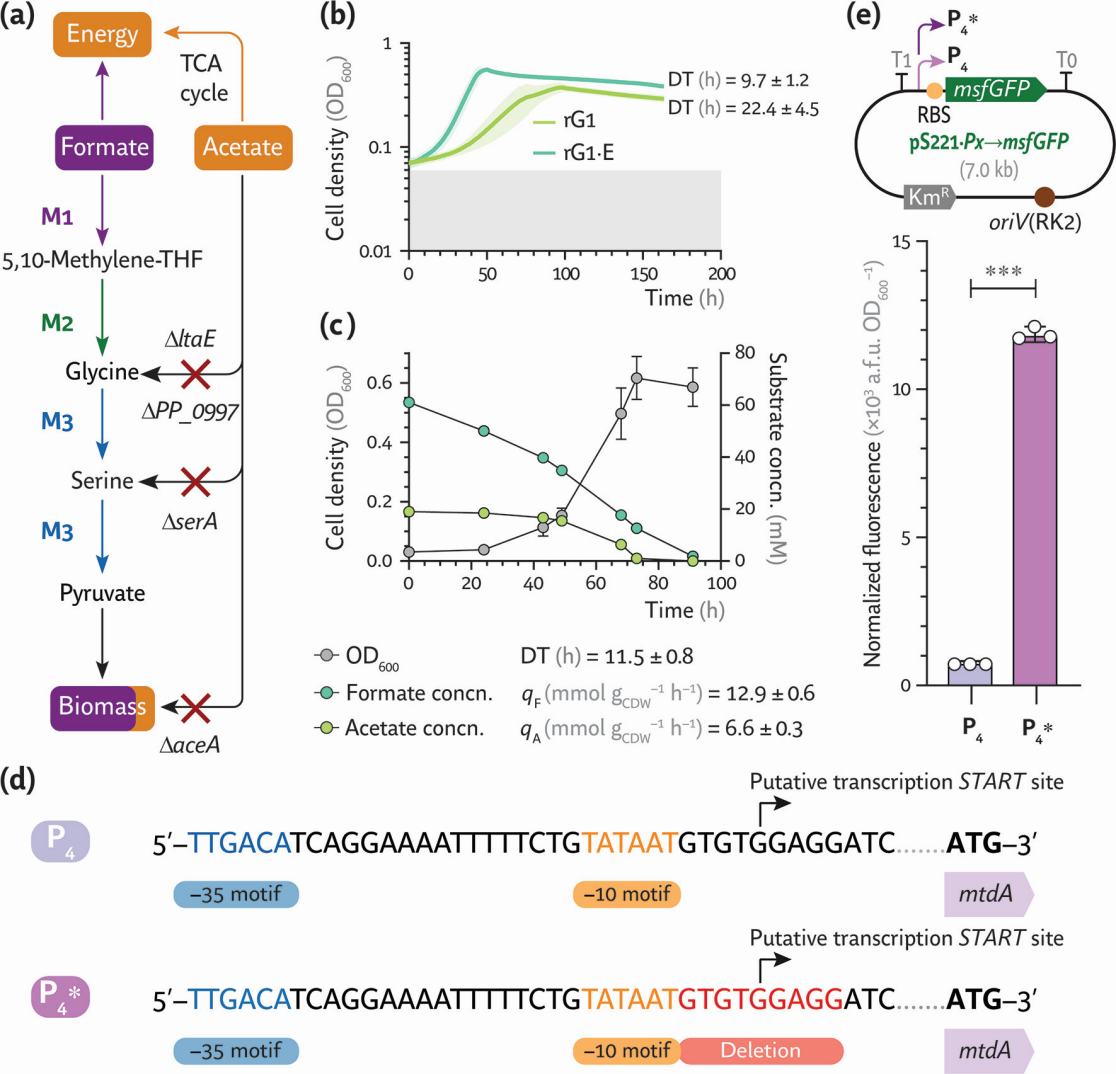
Engineering and evolution of *P. putida* toward assimilation of formate as the main carbon source. (**a**) Core metabolism engineered in strain rG1 for growth on formate as the main carbon substrate with acetate as the energy source. (**b**) Growth profile of *P. putida* rG1 and its evolved rG1·E derivative, isolated following ALE under mixotrophic conditions. Strains were cultivated in microtiter plates containing a minimal salt medium supplemented with 60 mM formate, 20 mM acetate, and 10% (vol/vol) CO_2_ in the headspace. Doubling times (DTs) were derived from average cell density values (estimated as the optical density at 600 nm, OD_600_) of four independent experiments. (**c**) Shaken-flask cultures of *P. putida* rG1·E incubated under mixotrophic conditions. Average values ± standard deviation for OD_600_, DT, and substrate concentration (concn.) are indicated for three independent experiments. CDW, cell dry weight. (**d**) Deletion of 9 bp in the synthetic P_4_ promoter (guiding the expression of module M1) upon ALE. (**e**) Structure of the transcriptional reporter plasmid used to assess the strength of the native (**P_4_**) and mutated (P_4_*) promoters. These promoter sequences were cloned such that they regulate the expression of the monomeric superfolder GFP gene (*msfGFP*). The normalized fluorescence (arbitrary fluorescence units [a.f.u.] divided by OD_600_) in cultures of *P. putida* EM42 carrying the corresponding reporter plasmid was measured after a 24-h incubation in lysogeny broth. Average values ± standard deviations are indicated for three independent experiments; the statistical significance was evaluated with the Student’s *t* test (***P < 0.001). Km^R^, kanamycin resistance determinant; RBS, ribosome binding site.

The starting point of this engineering strategy was *P. putida* rG1 (35), a derivative of the reduced-genome strain EM42 (38) in which the genes encoding the low-specificity L-threonine aldolase (LtaE, PP_0321), the bifunctional D-3-phosphoglycerate dehydrogenase/α-ketoglutarate reductase (SerA, PP_5155), and the putative GCS regulator (PP_0997) were eliminated (*P. putida* SLA, Fig. 1b), thereby blocking the canonical routes for glycine and serine biosynthesis while suppressing any native regulatory mechanism on the GCS (Fig. 2a). The activity of modules M1 and M2 of the rGlyP then restored glycine and serine production, respectively, from formate. Module M1, expressed from the medium-strength, constitutive P_4_ promoter (22), was integrated into the native *pha* locus while deleting the *pha* genes (PP_5002-PP_5008) by following a knock-in/knock-out strategy. This modification prevented carbon-diverting polyhydroxyalkanoate accumulation (39). Module M2 was overexpressed from the chromosome by promoter engineering (40), introducing a strong constitutive promoter (P_14g_) and translational coupler (BCD10) upstream of *gcvH-I* (PP_0989). Module M3 was overexpressed in a similar manner by placing the P_14g_ promoter and the BCD2 translational coupler upstream of the native *glyA-I* (PP_0322) and *glyA-II* (PP_0671) genes (*P. putida* cM1-3, Fig. 1b). Additionally, the deletion of *aceA* (PP_4116, which encodes isocitrate lyase) disrupted the glyoxylate shunt and prevented acetate assimilation. Consequently, in the resulting rG1 strain, formate is assimilated into biomass, while acetate provides NADH for energy conservation (Fig. 2a). The oligonucleotides and codon-optimized gene sequences used for engineering strain rG1 and its derivatives are listed in Tables S1 and S2, respectively.

Upon direct selection, *P. putida* rG1 barely grew under mixotrophic conditions, i.e., MSM supplemented with 60 mM formate, 20 mM acetate, and 10% (vol/vol) CO_2_ in the headspace, with perceptible turbidity arising only after 3–4 weeks of incubation. After six serial passages (corresponding to ca. 12 generations [41]) under the same conditions, *P. putida* rG1 grew slowly but steadily, displaying a doubling time (DT) of 22.4 ± 4.5 h (Fig. 2b). Prolonging ALE for an extra 35 generations led to the isolation of clone rG1·E. This evolved strain displayed a nearly halved DT compared with the parental strain (DT = 9.7 ± 1.2 h) and reached ca. 30% higher maximal cell density (optical density at 600 nm [OD_600_] = 0.52 ± 0.05) under mixotrophic conditions. Although microtiter plate cultures provided a rapid assessment of strain physiology, detailed quantitative parameters were needed. We then evaluated growth parameters and substrate (C_1_ and C_2_) consumption in shaken-flask cultures under the same medium composition and headspace conditions (Fig. 2c). The DT in these cultures was comparable to that observed in microtiter plates (DT = 11.5 ± 0.8 h), although the maximal cell density reached a slightly higher value (OD_600_ = 0.61 ± 0.03). Formate and acetate were co-consumed by strain rG1·E. Formate utilization started first, while acetate consumption began after 24 h and roughly coincided with the onset of exponential growth (Fig. 2c). This pattern indicates that acetate was used when the NADH demand rose alongside cell density. The specific rate of formate consumption (*q*_F_) was approximately twice as high as the specific rate of acetate consumption (*q*_A_), with *q*_F_ = 12.9 ± 0.6 mmol g cell dry weight (CDW)^–1^ h^–1^. These kinetic parameters indicate a large formate uptake required to supply biomass building blocks. Growth ceased once acetate was depleted, indicating that strain rG1·E relies on both C_1_ and C_2_ substrates for energy and precursor requirements.

Whole-genome sequencing revealed an alteration in the engineered promoter region of module M1 in clones with improved growth phenotypes after the short-term ALE experiment (Fig. 2d; Table S3). In particular, a 9-bp deletion (5′-GTGTGGAGG-3′) was identified next to the –10 motif of the P_4_ promoter. A plasmid-borne transcriptional reporter was constructed to assess the impact of this modification on gene expression. To this end, a *msfGFP* module (encoding the monomeric superfolder GFP [42], including a canonical ribosome binding site [RBS]) was cloned into the low-copy-number pSEVA221 vector (43–45), yielding plasmid pS221·*P_x_*→*msfGFP* (Fig. 2e). Next, the original (P_4_) and mutated (P_4_*) promoters were separately cloned in this reporter vector and introduced into *P. putida* EM42. Fluorescence levels were quantified after cultivation in lysogeny broth (LB) for 24 h, revealing that the 9 bp deletion near the TATA box of the P_4_ promoter increased the normalized msfGFP fluorescence by ca. 8-fold (Fig. 2e). Similar observations were made when the strains were grown in MSM containing 20 mM acetate as the sole carbon source (data not shown). These results suggest that high expression of the first module of the C_1_ assimilation pathway is essential to achieve rapid growth via the rGlyP when formate serves as the main carbon source.

Two other potentially significant polymorphisms were discovered in the genome of the evolved *P. putida* rG1·E strain (Table S3). One was a G→A single nucleotide polymorphism (SNP) in *folM* (PP_4632) that results in an alanine→threonine modification in position 89 of FolM. This bifunctional dihydrofolate reductase and dihydromonapterin reductase catalyzes the reduction of 7,8-dihydrofolate to THF, essential for the initial formate activation step. The other modification was a 3-bp deletion in *sdhA* (PP_4191), encoding the flavoprotein subunit of succinate dehydrogenase in the TCA cycle, which eliminated a leucine residue at position 444 of the enzyme. A reverse engineering strategy was used to test whether these polymorphisms, along with the 9-bp deletion in the P_4_ promoter, could contribute to the enhanced mixotrophic growth phenotype. Hence, the identified alterations in the P_4_ promoter (P_4_*), *folM*, and *sdhA* were introduced (reverse-engineered) into the non-evolved (naive) rG1 strain via homologous recombination (46), yielding strain rG1·RE (Table 1). As expected, *P. putida* rG1 grew very slowly in MSM containing formate and acetate, whereas robust mixotrophic growth was restored in strain rG1·RE, which had a DT = 16.5 ± 1.8 h (Fig. S1). While the DT remained slightly longer than that of *P. putida* rG1·E—indicating that other mutations in this strain may contribute to improving growth—this reverse engineering approach linked the mutations in the P_4_ promoter, *folM*, and *sdhA* to ca. 60% of the evolved rG1·E mixotrophic phenotype. The next step was to investigate the fate of carbon in these engineered strains, with a focus on the role of potentially competing pathways that may reroute key biomass precursors.

**TABLE 1:** Bacterial strains used in this study

Strain	Relevant characteristics^*a*^	Reference or source
*Escherichia coli*		
DH5α λ*pir*	Cloning host; F^– ^λ*^–^ endA1 glnX44*(*AS*) *thiE1 recA1 relA1 spoT1 gyrA96*(Nal^R^) *rfbC1 deoR nupG* Φ*80*(*lacZ*Δ*M15*) Δ(*argF-lac)U169 hsdR17*(*r_K_^–^ m_K_^+^*), λ*pir* lysogen	(47)
HB101	Helper strain; F^−^ λ^−^ *mcrB mrr hsdS20*(r_B_^−^m_B_^−^) *recA13 leuB6 ara-14* Δ(*proBA*)*2 lacY1 galK2 xyl-5 mtl-1 rpsL20*(Sm^R^) *glnV44*	(48)
*Pseudomonas putida*		
KT2440	Wild-type strain, derivative of *P*. *putida* mt-2 (49) cured of the TOL plasmid pWW0	(50)
EM42	Reduced-genome derivative of *P. putida* KT2440; Δ*PP_4329-PP_4397* (flagellar operon) Δ*PP_3849-PP_3920* (prophage I) Δ*PP_3026-PP_3066* (prophage II) Δ*PP_2266-PP_2297* (prophage III) Δ*PP_1532-PP_1586* (prophage IV) ΔTn7 Δ*endA-1* Δ*endA-2* Δ*hsdRMS* ΔTn*4652*	(38)
SLA	Derivative of *P. putida* EM42, Δ*serA* Δ*ltaE* Δ*aceA*	(35)
cM1-3	Derivative of *P. putida* EM42, Δ*serA* Δ*ltaE* Δ*aceA* Δ*PP_0997* P_14g_(BCD10)→*gcvH-I* Δ*PP_5002-PP_5008*::P_4_→*mtdA-fch-ftfL* from *M*. *extorquens*	(35)
rG1	Mixotrophic strain; derivative of *P. putida* EM42, Δ*serA* Δ*ltaE* Δ*aceA* Δ*PP_0997* P_14g_(BCD10)→*gcvH-I* Δ*PP_5002-PP_5008*::P_4_→*mtdA-fch-ftfL* from *M*. *extorquens* P_14g_(BCD2)→*glyA-I* P_14g_(BCD2)→*glyA-II*	(35)
rG1·E	ALE derivative of *P. putida* rG1	This study
rG2	Mixotrophic strain; derivative of *P. putida* rG1*,* Δ*crc* Δ*hexR* Δ*purT* P_EM7_(BCD10)→*gcvT-II* P_EM7_→*pntAA*	(35)
rG2·E	ALE derivative of *P*. *putida* rG2	This study
rG1·RE	Reverse-engineered derivative of *P*. *putida* rG1, P_4_*→*mtdA-fch-ftfL folM*^A89T^ *sdhA*^ΔL444^	This study
rG1·T	Derivative of *P*. *putida* rG1·E, Δ*thiO*	This study
rG1·TE	ALE derivative of *P. putida* rG1·T	This study
rG·F^P^	Synthetic formatotroph; derivative of *P*. *putida* rG2·E carrying plasmid pEMG (*fdh* from *Pseudomonas* sp. strain 101), Gm^R^	This study
rG·F^C^	Derivative of *P*. *putida* rG2·F^P^ cured of plasmid pEMG	This study
rG·F	Synthetic formatotroph; derivative of *P*. *putida* rG2·F^C^ carrying Tn*5*[P_14e_(BCD13)→*fdh*] as a chromosomal (*PP_4156*) insertion, Sm^R^	This study
rG·M	Synthetic methylotroph; derivative of *P*. *putida* rG2·F^C^ carrying Tn*5*[P_14e_(BCD2)→*^Cn^mdh*] as a chromosomal (*hutG*) insertion, Sm^R^	This study

^*a*^ Antibiotic markers: Gm, gentamicin; Km, kanamycin; Nal, nalidixic acid; and Sm, streptomycin.

### Glycine oxidation does not contribute to biomass formation of a mixotrophic *P*. *putida* strain

**Fig 3:**
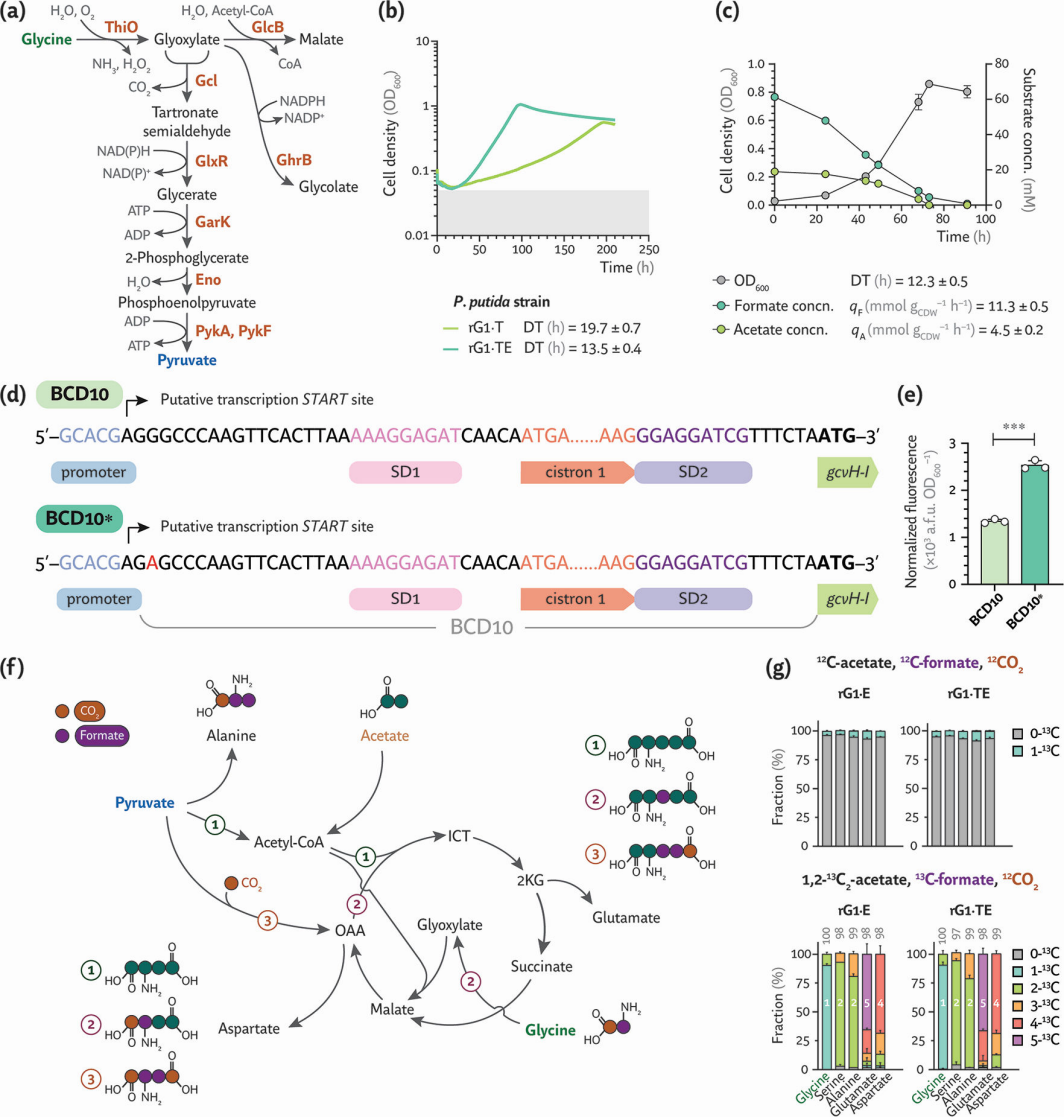
Exploring the role of glycine oxidase on formate assimilation by engineered *P. putida*. (**a**) Glycine could be oxidized by ThiO (FAD-dependent glycine/D-amino acid oxidase), ultimately yielding pyruvate. All enzymes depicted in the scheme are native to *P. putida*. (**b**) Growth profiles of mixotrophic *P. putida* strains with a Δ*thiO* deletion, analyzed before (rG1·T) and after ALE (rG1·TE). The strains were cultivated in microtiter plates in MSM with 60 mM formate, 20 mM acetate, and 10% (vol/vol) CO_2_ in the headspace; DTs were calculated from average cell density (OD_600_) values of four independent experiments. (**c**) Shaken-flask cultures of *P. putida* rG1·TE incubated under mixotrophic conditions. Average values ± standard deviation for OD_600_, DT, and substrate concentration are indicated for three independent experiments. (**d**) ALE-induced mutations in the bicistronic design (BCD) upstream of module M2. The mutated BCD contains a G→A transition downstream of the putative transcription *START* site. SD, Shine-Dalgarno sequence. (**e**) Normalized msfGFP fluorescence supported by the native (BCD10) and mutated (BCD10*) translational coupler elements in *P. putida* EM42 transformed with the corresponding reporter plasmids after a 24-h incubation in LB. Average values ± standard deviations are indicated for three independent experiments; the statistical significance was evaluated with the Student’s *t* test (***P < 0.001). (**f**) Labeling patterns in proteinogenic amino acids in ^13^C-tracer experiments to assess the contributions from (1) TCA cycle, (2) glycine oxidation and malate synthase, or (3) anaplerotic flux to mixotrophic growth. Acetyl-CoA, acetyl-coenzyme A; OAA, oxaloacetate; ICT, isocitrate; and 2KG, 2-ketoglutarate. (**g**) Labeling patterns in engineered *P. putida* strains grown with unlabeled formate and acetate (*upper panel*) or ^13^C-formate and 1,2-^13^C_2_-acetate (lower panel). The numbers above bars indicate the total fraction of ^13^C-labeled carbon atoms; values represent averages ± standard deviation of six independent experiments.

A FAD-dependent glycine/D-amino acid oxidase (ThiO, PP_0612) is encoded in the genome of *P. putida* and could potentially hamper formatotrophic growth by wasting electrons if glycine was oxidized to glyoxylate (Fig. 3a). In this oxidative branch of glycine processing, the amino acid initially undergoes oxidation to 2-iminoacetate, which then spontaneously converts to glyoxylate while releasing NH_3_ and generating H_2_O_2_ (51). The intermediate 2-iminoacetate also participates in thiamin biosynthesis. To improve growth on formate by preventing the formation of glyoxylate, we deleted *thiO* in the mixotrophic *P. putida* strain—supported by the observations previously made in engineered *C. necator* with DadA6, a D-amino acid dehydrogenase ortholog of ThiO (25). By deleting *thiO* in *P. putida* rG1·E, we generated strain rG1·T, which did not show a significant reduction in its DT upon cultivation in MSM supplemented with 60 mM formate and 20 mM acetate, with 10% (vol/vol) CO_2_ in the headspace, when compared to the naive rG1 strain.

We next employed ALE to further enhance mixotrophic growth performance in strain rG1·T. After a mere five passages (corresponding to ca. 10 generations), a clone designated rG1·TE exhibited an improved mixotrophic growth phenotype in microtiter plate cultures (Fig. 3b). The DT of this evolved variant decreased to 13.5 ± 0.4 h, and the biomass density increased 2-fold, reaching OD_600_ = 1.07 ± 0.11. As previously indicated for strain rG1·E, we carried out an assessment of growth parameters and substrate consumption under shaken-flask conditions using the same cultivation setup (Fig. 3c). Interestingly, the growth profile of rG1·TE largely resembled that of strain rG1·E. In this case, the *q*_A_ was about 2-fold lower than formate uptake (*q*_F_ = 11.3 ± 0.5 mmol g_CDW_^–1^ h^–1^), and both formate and acetate were nearly exhausted at the onset of the stationary phase. Although these findings suggest that ThiO is unlikely to supply substantial amounts of pyruvate for biomass, we submitted strain rG1·TE to whole-genome sequencing to uncover genetic changes responsible for the enhanced growth upon ALE. A substitution in the BCD10 translation coupler (G→A), close to the predicted transcription *START* site of *gcvH-I*, was detected in the evolved clone (Fig. 3d; Table S3). To elucidate the overall effect of this mutation on gene expression, *msfGFP* was expressed from the original BCD10 and the mutated BCD10* translational couplers in the parental *P. putida* EM42 strain, using the same reporter plasmid (pS221·*P_x_*→*msfGFP*, Fig. 2e) and cultivation conditions described above. The G→A transition boosted the normalized msfGFP fluorescence levels by ca. 2-fold (Fig. 3e), indicating that robust expression of the GCS components is important for formate assimilation during mixotrophic growth.

We next examined the metabolic fate of carbon atoms derived from formate and acetate in these strains by supplying a combination of ^12^CO_2_, ^12^C/^13^C-formate, and/or ^12^C/1,2-^13^C_2_-acetate, followed by analyzing the incorporation of these labeled feedstocks into proteinogenic glycine, serine, alanine, glutamate, and aspartate. Pyruvate and metabolites within the upper metabolic pathways should stem from formate and CO_2_ through the rGlyP, whereas TCA cycle intermediates could, in principle, originate from either formate or acetate (in case there is carbon assimilation through the TCA cycle). Hence, we supplemented 1,2-^13^C_2_-acetate to dissect the roles of cyclic flux around the TCA cycle, glycine oxidation to glyoxylate (ThiO) followed by assimilation through the activity of malate synthase (GlcB, PP_0356), and anaplerotic flux from pyruvate into the TCA cycle (Fig. 3f). If the TCA cycle proceeds cyclically, aspartate would mostly be labeled four times; if malate synthase or anaplerotic flux prevails, three- or two-times ^13^C-labeling would dominate the pattern. In general, no substantial differences were observed in the labeling patterns of strains rG1·E or its Δ*thiO* derivative described above (Fig. 3g). During cultivation of the strains in the presence of ^13^C-formate, 1,2-^13^C_2_-acetate, and ^12^CO_2_, 90% of glycine was labeled once, while 90% of serine and 80% of alanine were labeled twice. The fraction of glycine with two labeled carbons and serine with three labeled carbons can arise from either native formate oxidation to CO_2_ or decarboxylation steps in the TCA cycle. These activities release ^13^CO_2_, which can be reassimilated by the GCS. We also detected direct CO_2_ assimilation—since these strains were cultivated in a CO_2_-enriched atmosphere—via anaplerosis. The higher fraction of three-times labeled alanine, compared to serine, reflects a minor flux contribution from the TCA cycle into pyruvate. The four-times labeling of aspartate (70%) is consistent with cyclic flux within the TCA cycle and corresponds to the five-times labeled glutamate pool. Moreover, 20% and 10% of aspartate were labeled three and two times, respectively, in strains rG1·E and rG1·TE. The very minor portion of aspartate with three labeled carbons might stem from a synthase activity that relies on a non-annotated function or a promiscuous glycine oxidase or aminotransferase (52). Alternatively, the TCA cycle decarboxylation steps may release ^13^CO_2_, which is then recaptured by pyruvate carboxylase to form oxaloacetate from pyruvate (53). Overall, there was no significant difference in the glutamate labeling between strains rG1·E and rG1·TE (20% ± 3% and 22% ± 7% of glutamate containing four ^13^C atoms, respectively), verifying a negligible role for ThiO in glycine oxidation. Building on these results, we set out to establish formate as the sole carbon and energy source, as indicated in the next section.

### Engineering and characterization of a synthetic *P*. *putida* formatotroph

**Fig 4:**
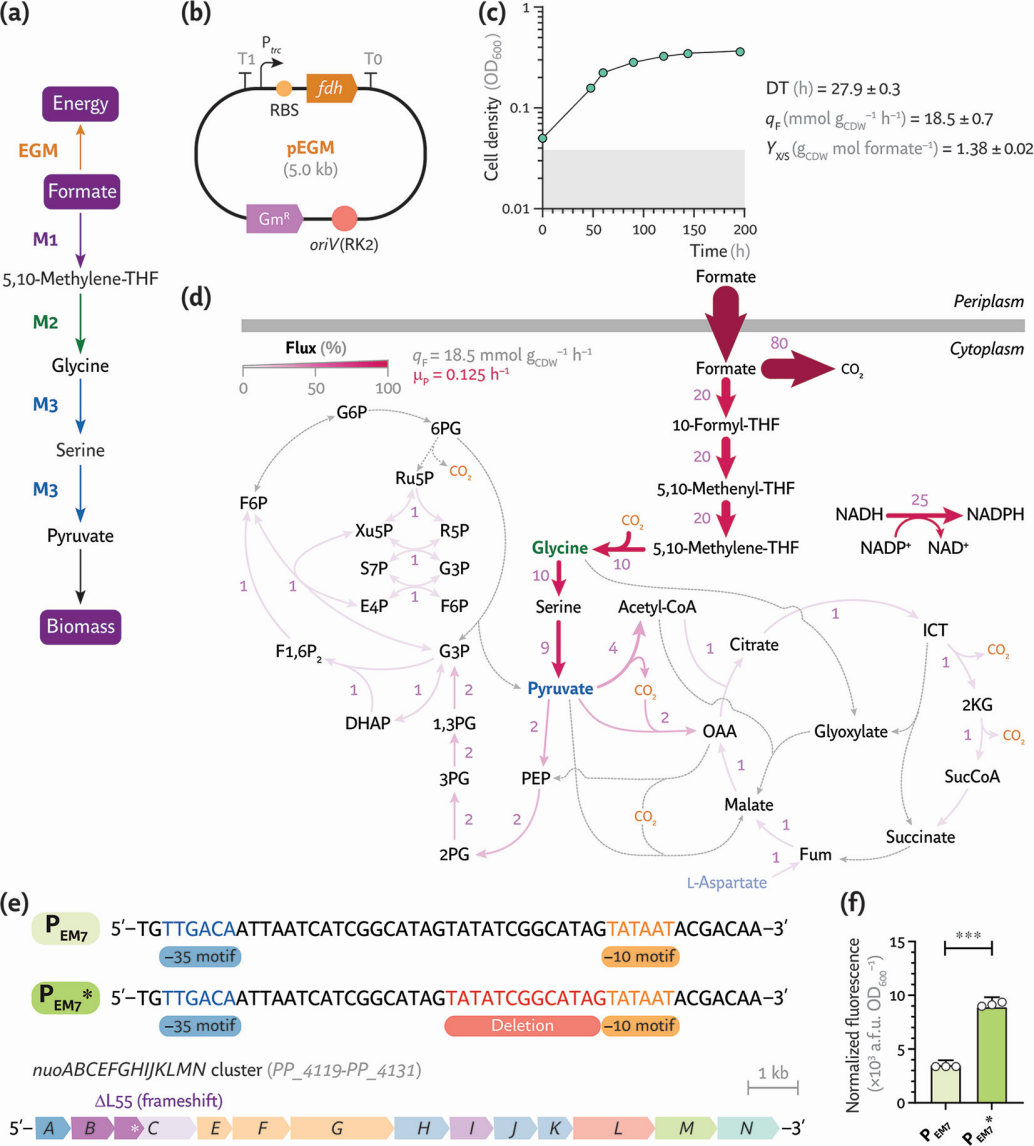
Expression of an energy generation module enables synthetic formatotrophy in *P. putida*. (**a**) Growth-coupled selection strategy to support formate assimilation as the sole carbon and energy source through the expression of the energy generation module. (**b**) Plasmid map of the EGM, constitutively expressing the *fdh* gene from *Pseudomonas* sp. strain 101. RBS, ribosome binding site; Gm^R^, gentamicin resistance determinant. (**c**) Shaken-flask cultures of the synthetic *P. putida* formatotroph (strain rG·F^P^) in MSM with 80 mM formate incubated under a 10% (vol/vol) CO_2_ atmosphere. Average values ± standard deviation for OD_600_, DT, substrate consumption, and biomass yield on substrate (*Y*_X/S_) are indicated for three independent experiments. (**d**) *In silico* modeling of formatotrophic growth in *P. putida* rG·F^P^. Fluxes, normalized to the specific formate consumption rate (*q*_F_ = 100), are represented as a percentage and visualized with scaled arrows. The predicted specific growth rate (*m*_P_) derived from the experimental *q*_F_ is indicated. Reactions with no flux are shown as gray dashed lines. Acetyl-CoA, acetyl-coenzyme A; 1,3PG, 1,3-bisphosphoglycerate; 2PG, glycerate-2-P; 3PG, glycerate-3-P; 2KG, 2-ketoglutarate; DHAP, dihydroxyacetone-P; E4P, erythrose-4-P; F6P, fructose-6-P; F1,6P_2_, fructose-1,6-bisphosphate; Fum, fumarate; G3P, glyceraldehyde-3-P; G6P, glucose-6-P; ICT, isocitrate; OAA, oxaloacetate; 6PG, 6-phosphogluconate; PEP, phosphoenolpyruvate; R5P, ribose-5-P; Ru5P, ribulose-5-P; S7P, sedoheptulose-7-P; SucCoA, succinyl-CoA; THF, tetrahydrofolate; and Xu5P, xylulose-5-P. (**e**) Key mutations detected in *P. putida* rG·F^P^ upon ALE. These modifications include a 13-bp deletion in the P_EM7_ promoter upstream of *pntAA* and a single nucleotide deletion in *nuoC* that leads to a premature stop codon. (**f**) Normalized fluorescence supported by the original (P_EM7_) and the mutated (P_EM7_*) promoters. The promoters were cloned in the reporter plasmid pS221·*P_x_*→*msfGFP*, transferred to *P. putida* EM42, and the fluorescence was measured after a 24-h incubation in LB. Average values ± standard deviations are indicated for three independent experiments; the statistical significance was evaluated with the Student’s *t* test (***P < 0.001).

Earlier metabolic designs relied on acetate to supply NADH for energy conservation; hence, the next goal was to enable formate to provide all carbon and energy needed for biomass formation (Fig. 4a). To this end, the *fdh* gene from *Pseudomonas* sp. strain 101 (54), encoding a NAD^+^-dependent formate dehydrogenase (Fdh), was placed under the constitutive P*_trc_* promoter in the low-copy-number pSEVA621 backbone, resulting in plasmid pEMG (Fig. 4b). Strain rG2 was also included in our experiments to broaden the range of bacterial *chassis* for testing synthetic formatotrophy (Table 1). This derivative of *P. putida* rG1 contains modifications predicted to bolster formate assimilation and biomass formation: overexpression of *gcvT-II* (PP_5194) from a P_EM7_(BCD10) element to enhance the GCS activity; deletion of the central carbon metabolism regulators Crc (PP_5292) and HexR (PP_1021) genes to alleviate potential bottlenecks in carbon assimilation; deletion of *purT* (PP_1457), encoding a phosphoribosyl-glycinamide formyltransferase that consumes formate and ATP to produce *N*^2^-formyl-*N*^1^-(5-phospho-β-D-ribosyl)glycinamide; and overexpression of the genes encoding the membrane-bound, NADP^+^-dependent pyridine nucleotide transhydrogenase by integrating the constitutive promoter P_EM7_ in front of *pntAA* (PP_0156) to promote NADPH formation for anabolism (35). When evaluated under mixotrophic conditions (i.e., MSM supplemented with 60 mM formate and 20 mM acetate, with 10% [vol/vol] CO_2_ in the headspace) upon a few selective passages, leading to *P. putida* rG2·E, the resulting strain exhibited kinetic parameters (DT = 14.9 ± 0.8 h) comparable to those of rG1·E.

Strains rG1·E, rG1·TE, and rG2·E were transformed with plasmid pEGM and cultivated in MSM containing 60 mM formate with 10% (vol/vol) CO_2_ in the headspace (i.e., under strict formatotrophic conditions). All strains carrying pEGM grew similarly after extended incubation (>1 month), whereas the corresponding control strains harboring the empty pSEVA621 vector did not. The growth phenotype improved following 28 passages (ca. 1 month) under formatotrophic conditions while incrementally raising the formate concentration to 80 mM, representing 65 generations. Fourteen clones were recovered from the ALE experiment, and formatotrophic growth was evaluated again in microtiter plate cultures with 80 mM formate in a CO_2_-enriched atmosphere. Five clones exhibited similar growth profiles, displaying DTs ~ 27–31 h (Fig. S2); these individual isolates were then examined under shaken-flask cultivation with formate as the sole carbon and energy substrate. The top-performing synthetic formatotroph, termed *P. putida* rG·F^P^, had a doubling time of 27.9 ± 0.3 h and entered stationary phase after ca. 100 h at an OD_600_ ~ 0.45 (Fig. 4c). The formatotrophic strain had a *q*_F_ = 18.5 ± 0.7 mmol g_CDW_^–1^ h^–1^, 1.4-fold higher than the formate uptake observed when acetate was supplied as the energy source. The biomass yield on the C_1_ substrate was *Y*_X/S_ = 1.38 ± 0.02 g_CDW_ mol formate^–1^ (Fig. 4c), below the theoretical yield value of 6.6 g_CDW_ mol formate^–1^ estimated through modeling with a *P. putida* genome-scale metabolic reconstruction tailored to include the rGlyP reactions. In this case, *in silico* flux distribution analysis, constrained by the experimentally determined *q*_F_, predicted a specific growth rate μ_P_ = 0.125 h^–1^ (Fig. 4d)—corresponding to a DT ~ 6 h. Under these conditions, the model assigned ca. 80% of the formate uptake to NADH-generating substrate oxidation via Fdh, whereas ca. 10% was routed through glycine toward biomass precursors.

Whole-genome sequencing of strain rG·F^P^ uncovered two key genetic changes that appear to underpin its formatotrophic phenotype. First, a 13-bp deletion (5′-TATATCGGCATAG-3′) in the spacer region of the constitutive P_EM7_ promoter upstream of *pntAA* gave rise to a truncated variant, which we termed P_EM7_* (Fig. 4e; Table S3). Second, a single-nucleotide deletion in *nuoC* (PP_4121) introduced a frameshift, eliminating a leucine residue at position 55 and creating a premature *STOP* codon downstream (Fig. 4e). As noted above, PntAB is the membrane-bound transhydrogenase that uses the H^+^-motive force to transfer reducing equivalents from NADH to NADP^+^, generating NADPH while oxidizing NADH (55). The EGM module supplies NADH through formate oxidation (Fig. 4a), whereas NADPH is essential for the reduction of 5,10-methenyl-THF to 5,10-methylene-THF and other biosynthetic reactions. Although *pntAB* was originally placed under P_EM7_ in an attempt to boost NADPH formation in strain rG2, the resulting transhydrogenase activity was apparently suboptimal. To quantify the effect of the deletion, the native and mutated P_EM7_ promoters were individually fused to *msfGFP* in the plasmid-borne transcriptional reporter (pS221·*P_x_*→*msfGFP*, Fig. 2e) and assayed in *P. putida* EM42 grown in LB medium for 24 h. The P_EM7_* variant increased normalized msfGFP fluorescence levels by ca. 2.5-fold (Fig. 4f), indicating higher promoter strength and, by extension, elevated transhydrogenase expression. Overexpression of the *pnt* genes from the unmodified promoter therefore seems to have failed in maintaining an optimal NADH/NAD^+^ and NADPH/NADP^+^ balance required for strictly formatotrophic growth. Consistent with this rationale, the mutation found in *nuoC* further emphasizes that keeping an appropriate NADH/NAD^+^ ratio—and, by extension, overall redox balance—is essential to support synthetic formatotrophy. NuoC forms part of complex I (NADH-quinone oxidoreductase), which oxidizes NADH and pumps H^+^ across the inner membrane (56). Truncation of NuoC after ca. one-fifth of its length (593 amino acids) is expected to disrupt or severely impair the NuoABCEFGHIJKLMN complex. Reduced activity of the respiratory complex I, in turn, would conserve cytosolic NADH (57), providing additional reducing power for PntAB (and potentially for other dehydrogenases and oxidoreductases) to provide NADPH and regenerate oxidized NAD^+^. This network-wide redistribution of reducing equivalents plausibly improves both formate oxidation and assimilation. However, we note that introducing the *nuoC* frameshift alone into strain rG2 did not immediately promote formatotrophic growth, indicating that improved C_1_-trophic performance arises from the combined action of P_EM7_* and the respiratory mutation—and possibly other secondary mutations (Table S3).

### Formate supplies the carbon skeleton of all proteinogenic amino acids in *P*. *putida* rG·F^P^

**Fig 5:**
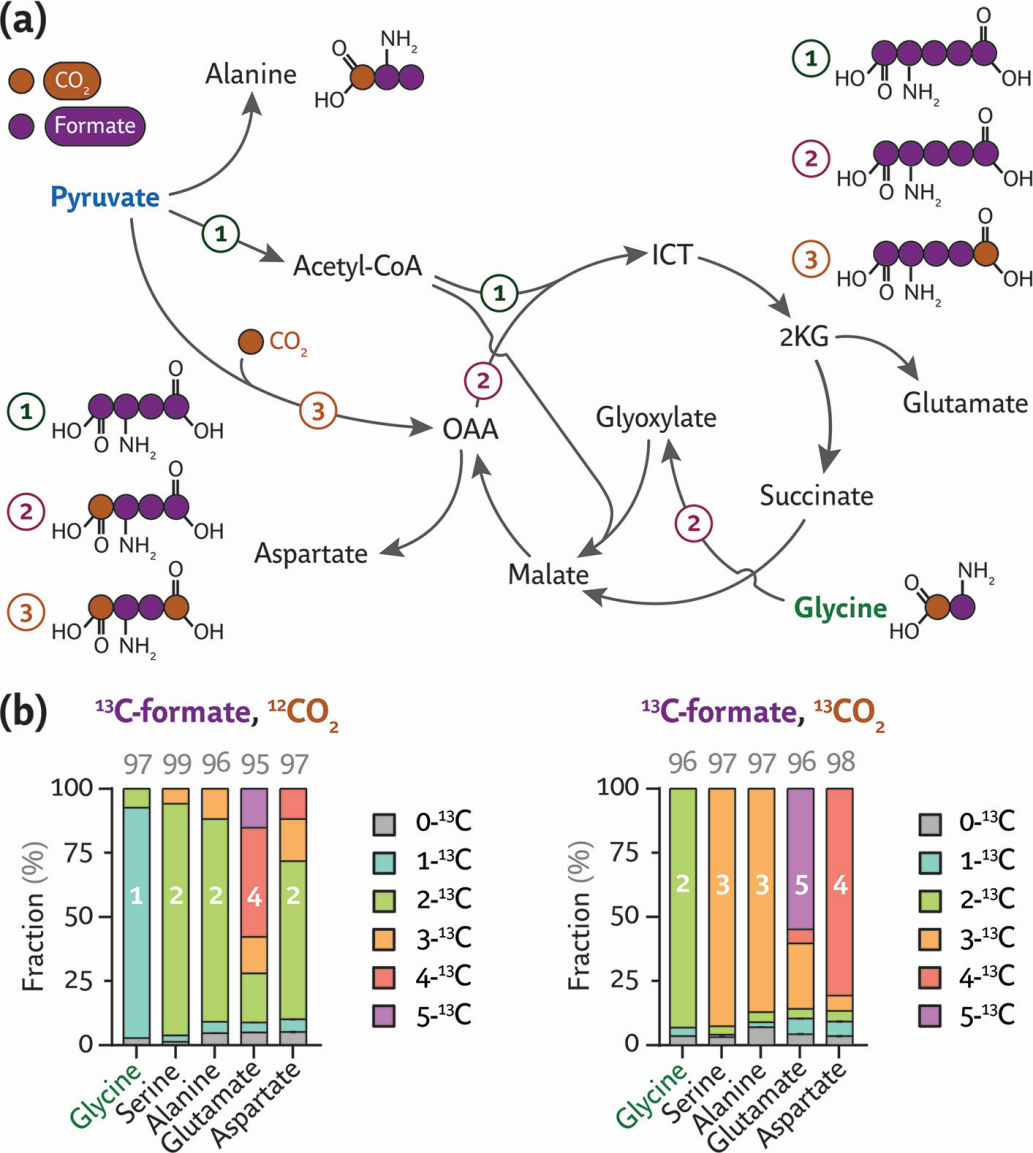
Labeling pattern of key proteinogenic amino acids underlying formatotrophic growth. (**a**) Labeling patterns in proteinogenic amino acids in ^13^C-tracer experiments to assess the contributions from (1) TCA cycle, (2) glycine oxidation and malate synthase, or (3) anaplerotic flux to formate assimilation in *P. putida* rG·F^P^. (**b**) Labeling patterns in engineered *P. putida* strains grown with labeled formate and unlabeled CO_2_ (left panel) or labeled formate and CO_2_ (right panel). The numbers above bars indicate the total fraction of ^13^C-labeled carbon atoms; values represent averages ± standard deviation of six independent experiments.

To map the origin and fate of all carbons in the synthetic formatotroph, the distribution of carbon atoms originating from formate and CO_2_ was assessed by feeding the cultures with mixtures of ^12/13^CO_2_ and ^13^C-formate. The incorporation of labeled ^13^C substrates into proteinogenic glycine, serine, alanine, glutamate, and aspartate was quantified as indicated for the mixotrophic strains. In the fully formatotrophic background, TCA cycle intermediates can mainly arise from three routes—i.e., closed TCA cycling, glyoxylate formation followed by malate synthase activity, or entry via pyruvate carboxylase and anaplerosis—each leaving a distinct isotopic fingerprint (Fig. 5a). When ^13^C-formate was paired with unlabeled (^12^C) CO_2_, 90% of glycine carried a single label and most serine (90%) and alanine (80%) bore two ^13^C labels (Fig. 5b), compatible with direct anaplerotic CO_2_ incorporation. A minor amino acid pool (corresponding to 7% of glycine, 6% of serine, and 12% of alanine) exhibited higher isotopic enrichment, attributable to recycling of ^13^CO_2_—released by formate oxidation and decarboxylations within the TCA cycle—subsequently reassimilated by the GCS. The aspartate pool displayed a dominant double-labeled isotopomer (62%), consistent with anaplerotic replenishment from pyruvate rather than full TCA cycle turnover. Glutamate labeling mirrored the aspartate isotopic distribution; ca. 50% of the pool was double-labeled, with the remainder split among tri- and tetra-^13^C-labeled amino acid species, indicating that 2-ketoglutarate is produced mostly after one passage through the oxidative branch of the TCA cycle, followed by reductive amination. Swapping ^12^CO_2_ by ^13^CO_2_ revealed the impact of CO_2_ fixation in the synthetic formatotroph (Fig. 5b). In these experiments, 93% of glycine now contained two ^13^C labels, serine and alanine were predominantly triple-labeled (93% and 87%, respectively), and 80% of aspartate together with 55% of glutamate carried four and five ^13^C atoms, respectively. The negligible pool of triple-labeled aspartate in ^12^CO_2_ experiments argues against a glyoxylate shunt contribution, indicating that ThiO-dependent glycine oxidation plays no significant role in catabolism in this strain. Collectively, these isotopomer patterns substantiate that the rGlyP supplies pyruvate from formate and CO_2_, while anaplerotic carboxylation replenishes oxaloacetate, limiting the need for full TCA cycling under formatotrophic conditions—in full agreement with the *in silico* flux distribution analysis (Fig. 4d). Carbon from both formate and CO_2_ is therefore efficiently captured by the synthetic formatotroph and routed into central metabolism for anabolism. Although the ^13^C labeling accounted for >96% of proteinogenic amino acids across all experiments, we note that complete labeling could not be achieved because *P. putida* rG·F^P^ underwent relatively few doublings during these growth experiments, leaving a small—yet consistently detectable—fraction of pre-existing unlabeled biomass.

### Mini-Tn*5*-mediated chromosomal integration of an EGM module supports a stable formatotrophic phenotype

**Fig 6:**
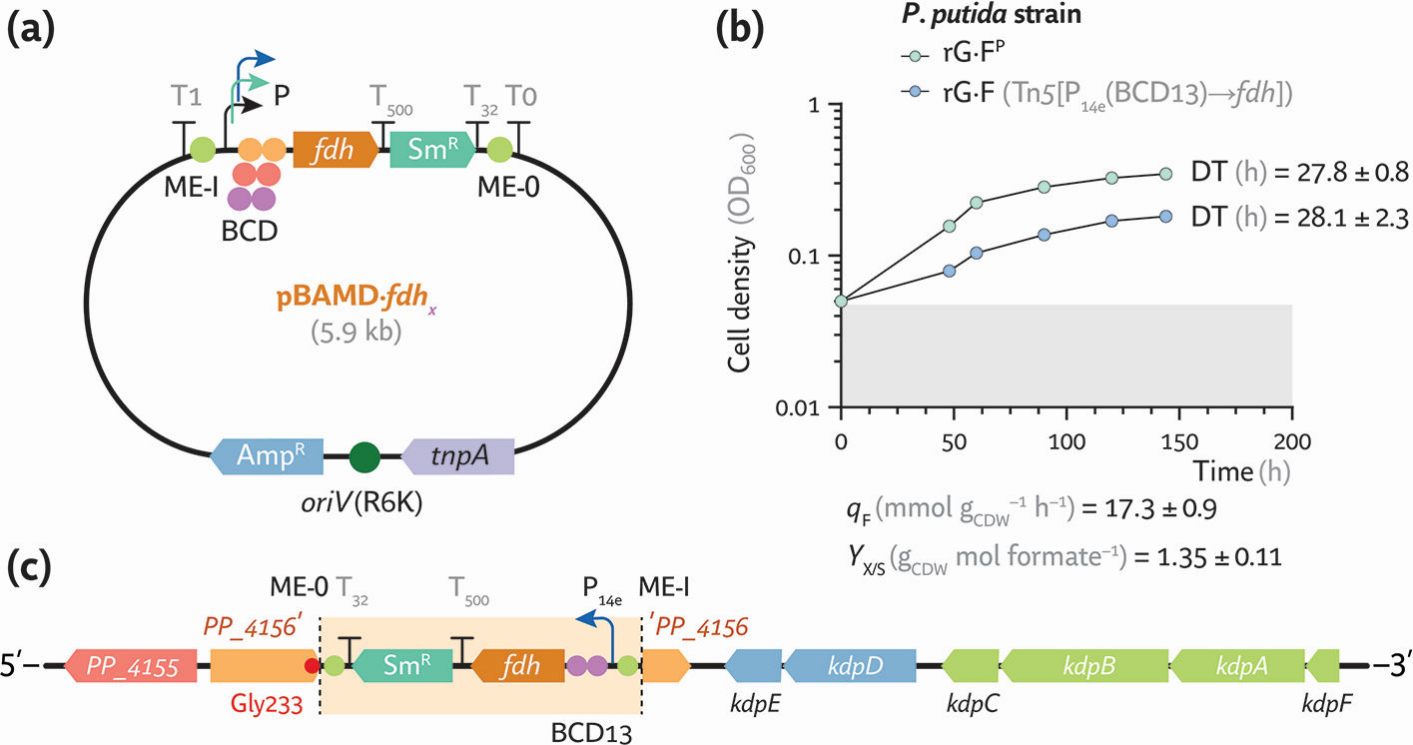
Construction of a stable synthetic *P. putida* formatotroph. (**a**) A mini-Tn*5* system was used for genomic integration of a randomized expression library for the EGM module. Amp^R^, ampicillin resistance determinant; Sm^R^, streptomycin resistance determinant; *tnpA*, gene encoding the hyperactive TnpA transposase; ME-I and ME-O, mosaic elements. (**b**) Growth profiles of formatotrophic *P. putida* strains carrying the EGM module either on a plasmid or integrated into the chromosome. Strains were incubated in shaken-flask cultures with MSM supplemented with 80 mM formate and 10% (vol/vol) CO_2_ in the headspace. Average values ± standard deviation for OD_600_, DT, substrate consumption, and biomass yield on substrate (*Y*_X/S_) are indicated for three independent experiments. (**c**) Genomic coordinates of the mini-Tn*5* module carrying P_14e_(BCD13)→*fdh*. Functional elements in the diagram are not drawn to scale.

To reduce the metabolic burden imposed by plasmid-borne expression of the EGM module (58) and sustain long-term, stable Fdh activity, *fdh* was integrated into the chromosome of *P. putida*. Although chromosomal insertion secures stable gene expression without continuous antibiotic selective pressure, identifying an optimal locus is challenging because transcriptional output varies with genomic context (59). Copy number differences near the origin of replication and local supercoiling, among other factors, are two well-established sources of this positional heterogeneity (60). Because the required Fdh levels could not be predicted *a priori*, we opted for an unbiased strategy based on the mini-Tn*5* transposon vector pBAMD1-4 (61). A small expression-cassette library was constructed by randomly combining three constitutive promoters (P_EM7_, P_14e_, and P_14g_) and three translational couplers (BCD13, BCD10, and BCD2) of increasing strength (62, 63). Each promoter-translational coupler pair was fused upstream of *fdh*, generating the pBAMD·*fdh*_x_ series of transposon vectors (Fig. 6a). Next, the auxiliary plasmid pEMG was removed from *P. putida* rG·F^P^ by serial passage under nonselective (LB) conditions, leading to strain rG·F^C^. The randomized plasmid pool was conjugated into *P. putida* rG2·F^C^, and transconjugants were first recovered on LB agar. Individual colonies were replica-plated onto MSM agar supplemented with 60 mM formate and streptomycin (Sm, the antibiotic resistance cassette present in the transposon module) and incubated under 10% (vol/vol) CO_2_. Only transconjugants carrying a functional Tn*5*[P*_x_*(BCD)*_y_*→*fdh*] insertion can oxidize and assimilate formate, providing stringent growth-coupled selection. Nine independent colonies displayed robust growth on formate after a second passage in the same medium, confirming a stable formatotrophic phenotype. The fastest-growing clone, designated *P. putida* rG·F, was evaluated in microtiter cultures alongside strain rG·F^P^, which carries *fdh* on plasmid pEGM (Fig. 6b). Both strains exhibited nearly identical exponential-phase kinetics; the DT of *P. putida* rG·F was 28.1 ± 2.3 h. The final cell density for the integrant formatotroph was ca. 15% lower than that of strain rG·F^P^. The substrate uptake (*q*_F_ = 17.3 ± 0.9 mmol g_CDW_^–1^ h^–1^) and biomass yield on the C_1_ substrate (*Y*_X/S_ = 1.35 ± 0.11 g_CDW_ mol formate^–1^, Fig. 6b) were, however, virtually indistinguishable from the plasmid-based control strain.

Whole-genome sequencing located the mini-Tn*5* element, carrying *fdh* under transcriptional control by the medium-strength P_14e_ promoter coupled with the weak BCD13 element (Fig. 6c), within *PP_4156*. This is a 906-bp long gene, encoding an uncharacterized LysR-type transcriptional regulator, a protein family widely involved in regulating metabolic and stress-related pathways. The transposon entered in reverse orientation after the codon encoding glycine at position 233 of the protein, truncating its *C*-terminal quarter. While functional studies specific to *PP_4156* are lacking, its sequence architecture, including a conserved helix-turn-helix DNA-binding domain and a predicted co-inducer-binding region, supports a regulatory role. A STRING-based network analysis revealed potential associations with other LysR regulators (*PP_2833*, *PP_3779*, and *PP_3934*), suggesting that PP_4156 may participate in broader transcriptional programs related to aromatic catabolism or environmental stress. Orthology analyses using OrtholugeDB identified conserved *PP_4156* homologs across *P. putida* strains BIRD-1, F1, and HB3267. Although no direct connection to synthetic C_1_ assimilation is evident, *PP_4156* may contribute to the cellular response to formate toxicity. Of note, *PP_4156* resides upstream of the genes encoding the KdpD/KdpE two-component system involved in maintaining K^+^ homeostasis (64) and downstream of *PP_4155*, predicted to encode an amine oxidase. The impact of disrupting PP_4156 remains to be established, yet the overall strain physiology indicates that potential effects linked to inserting the Tn*5*[P_14e_(BCD13)→*fdh*] module do not compromise formatotrophy while providing enough Fdh activity to support growth from a single *fdh* copy. Taken together, these findings demonstrate how random, Tn*5*-mediated chromosomal integration of the EGM followed by growth-coupled selection could be harnessed for synthetic formatotrophy, underscoring the utility of this workflow for pathway engineering—demonstrated by establishing methylotrophy in *P. putida* as explained below.

### Engineering a synthetic *P*. *putida* methylotroph

**Fig 7:**
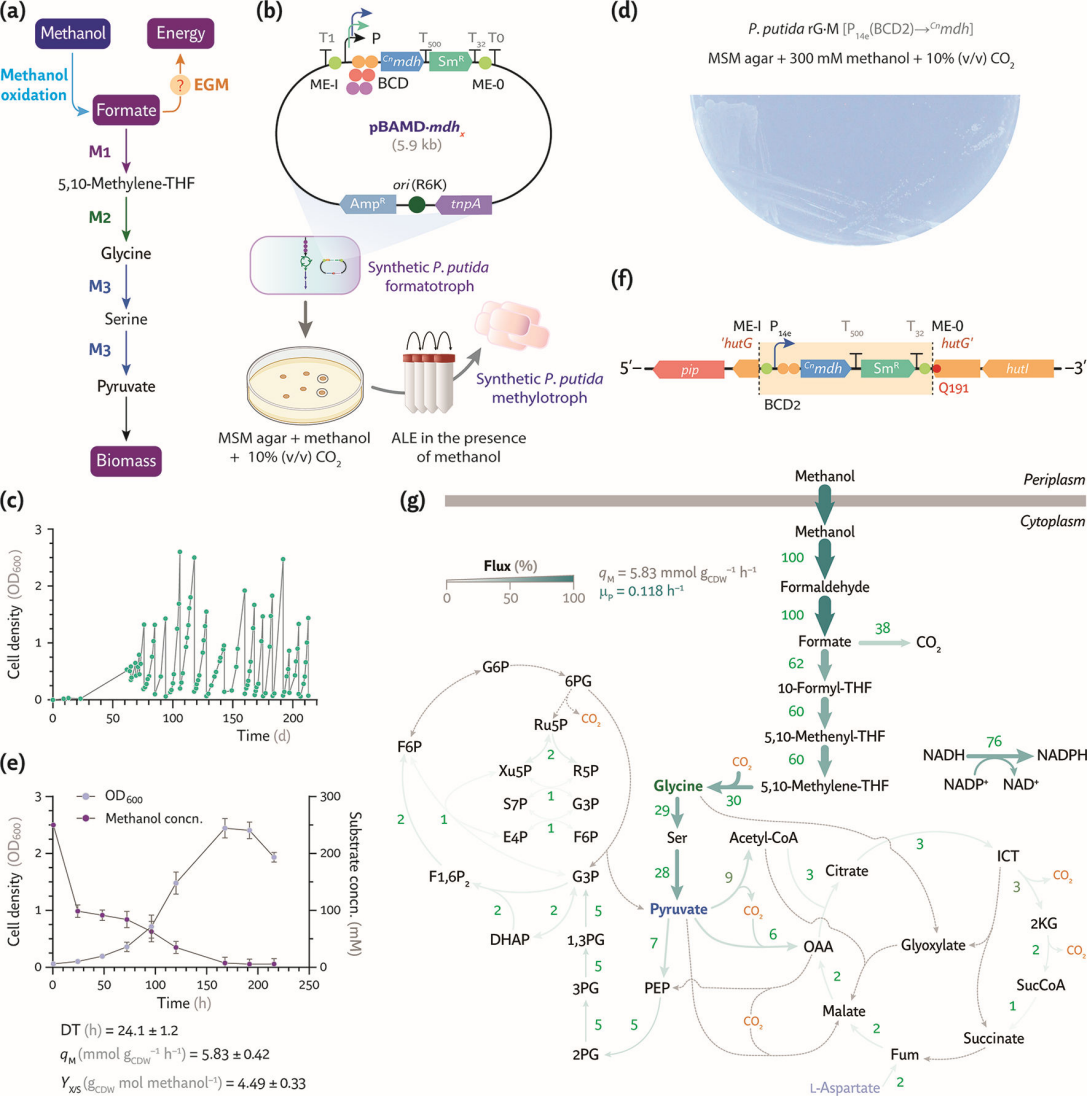
Construction of a stable synthetic *P. putida* methylotroph. (**a**) Growth-coupled selection strategy to support methanol assimilation as the sole carbon and energy source. In the resulting strain, the EGM no longer sustains energy conservation. (**b**) A mini-Tn*5* system enabled genomic integration of a NAD^+^-dependent methanol dehydrogenase gene from *C. necator* (*^Cn^mdh*). A randomized expression library was constructed as explained for *fdh*. (**c**) ALE trajectory of engineered *P. putida* rG·F^C^ carrying the methylotrophy transposon module randomly integrated in the chromosome. The ALE run began with MSM supplemented with 300 mM methanol and 20 mM acetate until growth was detected, followed by further dilutions in fresh MSM with 300 mM methanol and 10% (vol/vol) CO_2_ in the headspace. (**d**) Growth of *P. putida* rG·M on MSM agar supplemented with 300 mM methanol and 10% (vol/vol) CO_2_ in the headspace. (**e**) Shaken-flask cultures of the synthetic *P. putida* methylotroph (strain rG·M) in MSM with 300 mM methanol incubated under a 10% (vol/vol) CO_2_ atmosphere. Average values ± standard deviation for OD_600_, DT, specific methanol consumption rate (*q*_M_), and *Y*_X/S_ are indicated for three independent experiments. (**f**) Genomic coordinates of the mini-Tn*5* module carrying P_14e_(BCD2)→*^Cn^mdh*. Functional elements in the diagram are not drawn to scale. (**g**) *In silico* modeling of methylotrophic growth in *P. putida* rG·M. Fluxes, normalized to *q*_M_ = 100, are represented as a percentage and visualized with scaled arrows. The predicted specific growth rate (μ_P_) derived from the experimental *q*_M_ is indicated. Reactions with no flux are shown as gray dashed lines.

The rGlyP can sustain methylotrophic growth when alcohol oxidation delivers a steady supply of formate for carbon assimilation (Fig. 7a). Compared with direct formate oxidation, methanol oxidation supplies additional reducing equivalents (20)—thereby increasing ATP generation—and can render the auxiliary EGM dispensable while still preserving formate for biosynthesis. This thermodynamic advantage prompted us to replace formate oxidation with a highly active methanol oxidation step in the formatotrophic *chassis*. An optimized methanol dehydrogenase was selected to test the strategy. The engineered CT41 variant of the NAD^+^-dependent methanol dehydrogenase Mdh2 from *C. necator* N1 displays three amino acid substitutions that increase the *k*_cat_/*K*_*m*_ for methanol by 6-fold (65). The corresponding gene, *^Cn^mdh*, was cloned into the mini-Tn*5* vector pBAMD14 downstream of a set of synthetic constitutive promoters and BCD modules previously validated for chromosomal expression of *fdh* (Fig. 7b). As it was the case for *fdh*, the resulting expression library, numbering nine independent transposon constructs, is expected to encompass a broad spectrum of transcriptional and translational strengths—maximizing the chance of pinpointing a configuration that balanced methylotrophic growth, redox, and energy demands.

To test if methanol oxidation could provide all the electrons needed for energy conservation while supporting C_1_ assimilation, the transposon-based *^Cn^mdh* expression library was delivered into strain rG·F^C^ by conjugation. After overnight recovery, the mixture was plated on MSM agar containing 300 mM methanol and Sm and incubated for 3 weeks under 10% (vol/vol) CO_2_. Roughly 150 transconjugants emerged; these were purified twice under the same conditions and transferred to liquid MSM supplemented with 300 mM methanol under a CO_2_-enriched atmosphere. Once turbidity became evident (OD_600_ ~ 0.1), ALE was initiated to accelerate methylotrophic growth (Fig. 7c). Cultures were passaged into fresh medium for 225 days (corresponding to ca. 85 generations) whenever the OD_600_ readings exceeded a threshold value while maintaining the same methanol and CO_2_ concentrations. Biomass accumulation was negligible during the first month, after which exponential growth resumed. Growth improvement plateaued near generation 85, and individual colonies were isolated on MSM agar supplemented with 300 mM methanol, ensuring selection of clones capable of reproducible methylotrophic growth. A representative isolate of this bacterial population, designated *P. putida* rG·M, could grow on MSM agar plates containing methanol as the only carbon source when incubated in a CO_2_-enriched atmosphere (Fig. 7d).

The methylotrophic rG·M strain was further characterized in shaken-flask cultures in MSM containing 300 mM methanol under 10% (vol/vol) CO_2_, displaying a robust C_1_-trophic phenotype and ultimately attaining an OD_600_ of 2.5 ± 0.3 after ca. 150 h—substantially above the parental formate-assimilating control strain (Fig. 7e). The DT averaged 24.1 ± 1.2 h in these cultures, while the specific methanol consumption rate was *q*_M_ = 5.83 ± 0.42 mmol g_CDW_^–1^ h^–1^. Methanol disappeared rapidly during the first 24 h, consistent with preferential alcohol oxidation for NADH generation, and diminished more slowly thereafter, as intracellular formate accumulated for anabolic assimilation. The biomass yield on methanol for *P. putida* rG·M was *Y*_X/S_ = 4.49 ± 0.33 g_CDW_ mol methanol^–1^ (Fig. 7e), which is ca. 25% of the theoretical maximum under these conditions.

Whole-genome sequencing revealed that expression of the methanol oxidation cassette selected under these conditions is driven by the medium-strength P_14e_ promoter and the strong BCD2 translational coupler. The transposon is inserted in reverse orientation relative to *hutG* (PP_5029, *N-*formyl-L-glutamate deformylase) at the codon encoding the glutamine residue at position 191 (Fig. 7f). HutG is part of the histidine degradation via urocanate (66). Apart from this landing site for the Tn*5*[P_14e_(BCD2)→*^Cn^mdh*] module, the evolved and parental genomes were nearly identical (Table S3). We also identified an insertion sequence involving the 1,098-bp long *PP_4459* transposase within the *C*-terminal domain of a putative histidine kinase encoded by *PP_2683*, likely occurring during the early stages of mixotrophic evolution. PP_2683 is predicted to form a two-component system with YiaY (PP_2682), a NAD^+^-dependent methanol dehydrogenase (67). The *yiaY* gene is part of the *ped* cluster and is upregulated during exposure of *P. putida* to methanol (36). Disruption of this regulatory circuit probably favored methanol processing by the heterologous CT41 dehydrogenase instead of the native alcohol oxidation activities of *P. putida*.

Finally, *in silico* flux distribution analysis, constrained by the experimentally determined *q*_M_, predicted a specific growth rate μ_P_ = 0.118 h^–1^ for the synthetic methylotroph (Fig. 7g). In this case, the model assigned ca. 38% of the methanol uptake to substrate oxidation to CO_2_, whereas ca. 30% was routed through glycine toward biomass precursors.

## DISCUSSION

In this study, we expanded the portfolio of synthetic bacteria that assimilate C_1_ substrates by engineering the metabolically versatile *P. putida* to grow on formate and methanol. We selected *P. putida* as the host due to intrinsic metabolic features that could facilitate synthetic C_1_ assimilation. Since our approach relied on a stepwise transition from mixotrophic to fully C_1_-trophic metabolism, the ability to supply energy and reducing power at each stage was critical to sustain our engineering strategy. *P. putida* is known for its high rates of catabolic NADPH turnover, which can support increased anabolic demands. Moreover, its native central carbon metabolism, structured around the EDEMP cycle, may favor a robust supply of biomass precursors—unlike the more linear catabolic pathways predominant in other industrially relevant bacteria, e.g., *E. coli*. Based on these considerations, the first set of *P. putida* strains harboring the rGlyP modules M1, M2, and M3 relied on formate as the carbon source and acetate for energy. Subsequent rounds of rational and evolutionary (ALE) engineering under mixotrophic conditions produced strain derivatives with markedly improved C_1_-trophic growth, underscoring the value of growth-coupled selection together with iterative design-build-evolve cycles for pathway optimization (68, 69).

During the first stage of ALE, two promoter-region alterations proved key to establishing formatotrophy. A single-base deletion in the discriminator sequence immediately downstream of the TATA box of the synthetic P_4_ promoter, together with a point mutation just beyond the predicted transcription *START* site of *gcvH-I*, boosted transcription of modules M1 and M2. The discriminator sequence and the nucleotides flanking the transcription *START* site dictate how efficiently RNA polymerase transcribes a given promoter (70, 71); shortening this region possibly facilitated promoter escape and strengthened gene expression (72). Reintroducing both mutations in the ancestral *P. putida* strain reproduced the adaptive phenotype, suggesting that high expression of the genes encoding the GCS and serine biosynthesis enzymes is essential for robust formate assimilation.

^13^C-Tracer experiments substantiated complete routing of the C_1_ feedstock into central metabolism, as the isotopic label accumulated in pyruvate and entered the TCA cycle through anaplerotic flux. By contrast, the native glycine oxidase—an enzyme that contributes substantially to formate assimilation in engineered *C. necator* (25)—made a negligible contribution in the engineered *P. putida*, indicating that the heterologous rGlyP dominates carbon assimilation through pyruvate once the expression of the cognate modules is properly balanced. Building on these results, we constructed the first fully formatotrophic *P. putida* strain by installing an EGM consisting of the metal-independent Fdh from *Pseudomonas* sp. strain 101—native formatotrophs rely mainly on metal-dependent dehydrogenases (73, 74). Native molybdenum-dependent Fdh variants encoded in the genome of *P. putida* (FmdEFGH, PP_2183-PP_2186) failed to support growth even when the corresponding genes were overexpressed from the chromosome (data not shown), suggesting that cofactor imbalance or catalytic inefficiency may limit their activity under oxic formatotrophic conditions.

Further evolution on formate selected two additional mutations that rewired redox metabolism toward synthetic C_1_ assimilation. The first, a deletion in the spacer region of the synthetic promoter guiding the expression of *pntAB*, shortened the spacer to the optimal 17-bp length typical of high-strength *E. coli* promoters (75). This SNP markedly increased transcription of the membrane-bound transhydrogenase genes. PntAB normally supplies approximately 40% of the NADPH required for biomass formation during aerobic *E. coli* cultivations (76); the repeated appearance of *pntAB*-activating mutations in independent formatotrophic lineages, including *E. coli* (22, 77), suggests that NADPH turnover also constrains anabolic rGlyP flux in *P. putida*. Enhanced transhydrogenase activity simultaneously regenerates NAD^+^, which is consumed by Fdh—possibly harmonizing the NADH/NAD^+^ and NADPH/NADP^+^ pools. The second mutation introduced a premature *STOP* codon in the peripheral C and D oxidoreductase subunits of the NADH dehydrogenase I complex. Truncations at homologous residues in *E. coli* are known to drastically diminish complex I assembly and activity (78); a similar reduction in NADH oxidation would raise the intracellular NADH/NAD^+^ ratio and drive transhydrogenation toward NADPH formation. Together, these changes improved the biomass yield to ca. 1.4 g_CDW_ mol formate^–1^—substantial for a *Pseudomonas chassis*, yet still below the 3.3 g_CDW_ mol formate^–1^ achieved by an evolved *E. coli* strain engineered with the rGlyP (26). From a broader perspective, strain rG·F compares favorably to other formatotrophs, both natural and synthetic (Table 2), and the current DT and biomass yields indicate headroom for further optimization.

**TABLE 2:** Growth properties of selected native and synthetic formatotrophs and methylotrophs^*a*^

C_1_-trophic mode and bacterial host	Pathway	Native/synthetic	*Y*_X/S_ (g_CDW_ mol substrate^– 1^)	DT (h)	Reference
Formatotrophy					
*C. necator*	CBB cycle	Native	2.9	5	(25)
*C. necator*	rGlyP	Synthetic	2.6	12	(25)
*C. necator*	rGlyP	Synthetic	3.2	11.4	(79)
*E. coli*	rGlyP	Synthetic	2.3	8	(22)
*E. coli*	rGlyP	Synthetic	3.3	6	(26)
*E. coli*	Serine-threonine cycle	Synthetic	N.R.	10	(77)
*P. putida* rG·F	rGlyP	Synthetic	1.35 ± 0.11	28.1 ± 2.3	This study
Methylotrophy					
*M. extorquens*	Serine cycle	Native	12.8 ± 1.6	3	(80, 81)
*Bacillus methanolicus*	RuMP cycle	Native	15.6 ± 2.7	1.73 (50°C) 5 (37°C)	(82, 83)
*Methylobacillus flagellatus*	RuMP cycle	Native	16	1 (37°C)	(84)
*E. coli*	RuMP cycle	Synthetic	12.5	3.5	(85)
*E. coli*	RuMP cycle	Synthetic	N.R.	4.3	(86)
*E. coli*	rGlyP	Synthetic	4.2 ± 0.2	54.9 ± 5.5	(22)
*P. putida* rG·M	rGlyP	Synthetic	4.49 ± 0.33	24.1 ± 1.2	This study

^*a*^ CBB cycle, Calvin-Benson-Bassham cycle; RuMP cycle, ribulose monophosphate cycle; and N.R., not reported.

We also established the first synthetic *P. putida* methylotroph. Overproduction of a mutant version of the NAD^+^-dependent Mdh2 dehydrogenase from *C. necator* enabled methanol oxidation, a circumstance previously exploited for mixotrophic metabolism (87). The native genome provides both pyrroloquinoline quinone (PQQ) biosynthetic genes and several PQQ-dependent alcohol dehydrogenases (88), yet their involvement in synthetic methylotrophy through the rGlyP—if any—is not fully clear. Substituting a PQQ-linked Mdh could overcome the kinetic limitation of NAD^+^-dependent alcohol oxidation (89), albeit at the expense of reduced biomass yield, due to the lower thermodynamic efficiency of PQQ-mediated electron transfer. Indeed, the kinetic constraints of methanol dehydrogenases are likely to contribute to the prolonged adaptation of strain rG·M to methanol-dependent growth. Nevertheless, the synthetic *P. putida* methylotroph grew faster and had a higher biomass yield on methanol than a similarly engineered *E. coli* methylotroph (Table 2), with a lag phase comparable to that of natural methylotrophs. Nevertheless, the kinetic parameters remain below those reported for native methylotrophs and *E. coli* strains equipped with the ribulose monophosphate cycle, highlighting scope for further optimization. In this sense, automated ALE pipelines could help accelerate strain optimization (68).

The spectrum of beneficial mutations uncovered here—spanning promoter architecture, redox-cofactor balance, and respiratory chain remodeling—highlights the power of growth-coupled selection and ALE to pinpoint levers that are difficult to predict *a priori*. In parallel, we demonstrated that Tn*5*-mediated random chromosomal integration, followed by selection under the desired growth regime, provides a rapid route to identify genomic landing pads that support high-level, stable expression without the burden brought about by plasmid maintenance. The synergy between rational pathway assembly and evolution-guided fine-tuning thus offers a versatile blueprint for constructing efficient formatotrophic and methylotrophic production hosts. Extending these principles to additional platform organisms and pairing C_1_ assimilation with diverse product pathways could anchor a sustainable bioeconomy rooted in formate, methanol, and related feedstocks generated from CO_2_ and renewable energy (90, 91).

## MATERIALS AND METHODS

### Chemicals and reagents

Chemicals were purchased from Sigma-Aldrich Co. (St. Louis, MO, USA) unless otherwise indicated, and oligonucleotides were synthesized by Integrated DNA Technologies Inc. (Coralville, IA, USA). DNA sequencing was performed at Eurofins Genomics Europe GmbH (Ebersberg, Germany). All primers and codon-optimized gene sequences used in this study are listed in Tables S1 and S2.

### Bacterial strains, media composition, and culture conditions

All bacterial strains and plasmids used in this study are listed in Table 1. *Escherichia coli* DH5α λ*pir* and HB101 were adopted as cloning and helper hosts; reduced-genome *P. putida* EM42 was selected for engineering formatotrophy and methylotrophy. Lysogeny broth (LB; 10 g L^−1^ tryptone, 5 g L^−1^ yeast extract, and 10 g L^−1^ NaCl) and minimal salt medium (92) were used for all cultivations. MSM contained 3.88 g L^−1^ K_2_HPO_4_, 1.63 g L^−1^ NaH_2_PO_4_, 2 g L^−1^ (NH_4_)_2_SO_4_, and 0.1 g L^−1^ MgCl_2_∙6H_2_O, with initial pH adjusted at 7.0. MSM was supplemented with a trace elements solution (10 mg L^−1^ ethylenediaminetetraacetic acid, 2 mg L^−1^ ZnSO_4_∙7H_2_O, 1 mg L^−1^ CaCl_2_∙2H_2_O, 5 mg L^−1^ FeSO_4_∙7H_2_O, 0.2 mg L^−1^ Na_2_MoO_4_∙2H_2_O, 0.2 mg L^−1^ CuSO_4_∙5H_2_O, 0.4 mg L^−1^ CoCl_2_∙6H_2_O, and 1 mg L^−1^ MnCl_2_∙2H_2_O) (93) and the carbon sources indicated in the text. When needed, ampicillin (Amp), gentamicin (Gm), and Sm were supplied at 100, 10, and 100 μg mL^−1^, respectively.

For standard mixotrophic growth, overnight (18 h) LB cultures were diluted 100-fold to inoculate a preculture in 5 mL of MSM supplemented with the corresponding additives and carbon sources indicated in the text. The precultures were incubated under a CO_2_-enriched atmosphere in a 50 mL culture tube at 30°C and 200 rpm. The biomass from these precultures was washed with fresh MSM to inoculate the main culture in 96-well microtiter plates, test tubes, or baffled 250-mL Erlenmeyer flasks (shaken-flask cultures) with the appropriate additives. For 96-well microtiter plate cultures, 150 µL of the washed cell suspended at OD_600_ = 0.05 was covered with 50 µL of mineral oil to prevent evaporation and incubated with orbital shaking in a Synergy H1 or ELx808 microtiter plate reader (BioTek Instruments Inc., Winooski, VT, USA). Test tube and shaken-flask cultivations were conducted in 5 or 50 mL of MSM medium, respectively, incubated at 30°C and 200 rpm. The specific growth rate (*μ*, in h^–1^) was calculated during exponential growth by linear regression on the average OD_600_ values (94); doubling times (in h) were estimated according to DT = *ln*(2)/μ.

### Construction of plasmids and strain engineering

All plasmids used in this study were constructed using uracil-excision (USER) cloning, unless otherwise indicated. DNA fragments were amplified with Phusion *U* Hot Start DNA polymerase (ThermoFisher Scientific Co., Waltham, MA, USA) according to the manufacturer’s recommendations using uracil-containing primers. Backbones were further digested with *Dpn*I prior to mixing 100 ng of each PCR fragment with 1 μL of *Dpn*I-treated plasmid and 1 μL of USER enzyme (New England BioLabs, Ipswich, MA, USA) in a final volume of 10 μL (95). The reaction was incubated for 30 min at 37°C, followed by a temperature decrease during 3 min (from 28°C to 20°C, 2°C per step) and incubation at 10°C for at least 10 min. Chemically competent *E. coli* DH5α λ*pir* cells (Table 1) were transformed with 5 μL of the USER mix, and, upon recovery, the cell suspension was plated onto LB agar plates containing the corresponding antibiotic(s).

Suicide plasmids for gene deletions and insertions were based on vector pGNW2 [*ori*(R6K), Km^R^ (96)], bearing homologous regions needed for chromosomal integration. The corresponding plasmids were individually delivered into the host by electroporating 500 ng of DNA into 50 µL of freshly prepared *P. putida* electrocompetent cells and washed three times with 300 mM sucrose. Electroporation was performed with a Gene Pulser XCell (Bio-Rad, Hercules, CA, USA) set to 2.5 kV, 25 µF capacitance, and 200 Ω resistance in a 2-mm gap cuvette. In some cases, plasmids were transferred by triparental conjugation, using *E. coli* HB101 (Table 1) carrying either plasmid pRK600 (97) or pRK2013 (98) as a helper strain. The recipient *P. putida* strain, the helper *E. coli* strain, and the *E. coli* donor strain were mixed, plated on a warm LB plate, and incubated at 30°C for 24 h before isolating individual clones by dilution streaking. In all cases, positive co-integration events were selected and further transformed with pQURE6·H, a conditionally replicative plasmid bearing the meganuclease I-*Sce*I gene (99) via electroporation. The homing I-*Sce*I meganuclease cuts the pGNW2 sequence co-integrated within the chromosome, forcing a second homologous recombination event. Cells were recovered in 1 mL of LB supplemented with 2 mM of 3-methylbenzoate (3-*m*Bz) for at least 3 h at 30°C and plated onto LB agar containing the corresponding antibiotic(s) added with 1 mM 3-*m*Bz to induce plasmid replication and I-*Sce*I expression. Positive clones were identified by colony PCR, verified by DNA sequencing, and cured from the resolving plasmid by serial dilution under non-selective (LB) conditions.

A transcriptional reporter plasmid was constructed by cloning the *msfGFP* gene into the low-copy-number plasmid pSEVA221, carrying an *oriV*(RK2), a Km^R^ cassette, and a standard RBS (44). The resulting plasmid, termed pS221·P*_x_*→*msfGFP*, was used for promoter probing. Whenever needed, the regulatory sequences in this reporter system were exchanged by other functional elements using the QuikChange Lightning site-directed mutagenesis kit (Agilent Technologies Inc., Santa Clara, CA, USA). Briefly, the entire plasmid backbone was amplified with forward and reverse primers containing the promoter sequence (and BCDs, when relevant) to be tested. The resulting amplicon was restricted with *Dpn*I (ThermoFisher Scientific Co.), phosphorylated, and ligated into vector pS221·P*_x_*→*msfGFP*. Phosphorylation and ligation were performed using T4 polynucleotide kinase and T4 DNA ligase (ThermoFisher Scientific Co.), respectively, according to the manufacturer’s protocols. Positive clones were identified by colony PCR and verified by DNA sequencing.

### Mini-Tn*5*-assisted integration of metabolic modules into the bacterial chromosome

Plasmid pBAMD1-4 [mini-Tn*5* transposon, *ori*(R6K), Amp^R^ Km^R^ (57)] was used as the backbone to construct small expression libraries for *fdh* and *^Cn^mdh*. In each case, a set of synthetic promoters and BCDs was randomly assembled into the transposon module together with the corresponding dehydrogenase gene. The NEBuilder HiFi DNA assembly master mix (New England BioLabs) was used to assemble the DNA fragments obtained by amplification with Phusion Hot Start DNA polymerase (ThermoFisher Scientific Co.) following the manufacturer’s specifications. Next, chemically competent *E. coli* DH5α λ*pir* cells were transformed with 5 μL of the assembled mix, and, upon recovery, the cell suspension was plated onto LB agar plates containing the corresponding antibiotics. The plasmid library (termed pBAMD·*fdh_x_* [Fig. 6a] and pBAMD·*^Cn^mdh_x_* [Fig. 7b]) was delivered by tri-parental mating into the specific *P. putida* strains indicated in the text. The next day, the biomass was streaked on an MSM agar plate containing the adequate carbon source (60 mM formate or 300 mM methanol) and Sm followed by incubation at 30°C and 10% (vol/vol) CO_2_ in the headspace until individual colonies were detected. These colonies were further isolated and purified twice under the same selective conditions prior to phenotypic and genotypic characterization.

### ALE experiments

Overnight cultures in LB were diluted 100-fold into 5 mL of MSM containing 20 mM glucose or 60 mM acetate, 60 mM formate or 300 mM methanol, and incubated with 10% (vol/vol) CO_2_ in the headspace. The chosen carbon source matched the phenotype under selection—i.e., formatotrophy or methylotrophy. Cells from these precultures were harvested by centrifugation, washed twice with fresh MSM, and used to inoculate ALE cultures at an initial OD_600_ of 0.05. ALE was carried out in 12 mL glass tubes containing 3 mL of MSM supplemented with the same carbon substrates and additives. Cultures were incubated at 30°C at 200 rpm. Upon reaching the stationary phase, each culture was transferred to fresh medium by diluting the suspension to an OD_600_ ~ 0.05–0.1, and this passaging continued daily. OD_600_ was monitored directly in the tubes with an automated OD_600_ DiluPhotometer (Implen GmbH, Munich, Germany). ALE proceeded until the specific growth rates and final biomass no longer improved. To obtain individual clones, evolved populations of *P. putida* were streaked on MSM agar plates containing the relevant substrates and incubated under the same selective conditions (40). Single colonies were re-streaked twice on LB agar to rule out physiological adaptation, followed by two additional rounds of purification on selective MSM agar before further characterization.

### Whole-genome sequencing of *P*. *putida* strains

Genomic DNA was extracted from 2 mL overnight LB cultures of isolated *P. putida* clones with the PureLink Genomic DNA Mini kit (ThermoFisher Scientific Co.). Library preparation, sequencing, and initial quality control were carried out by Novogene Co. Ltd. (Cambridge, UK). Genomic libraries were sequenced on an Illumina NovaSeq 6000 platform in 150 bp paired-end mode. Raw reads were paired, quality-trimmed with the BBDuk plugin, and digitally normalized. High-quality reads were then assembled against the *P. putida* EM42 reference genome in Geneious Prime 2021.1.1 (Dotmatics, Boston, MA, USA). Single-nucleotide polymorphisms and small insertions or deletions were identified with the Geneious variant-calling workflow using a minimum variant frequency threshold of 0.50. Polymorphic sites were retained only when absent from the corresponding pre-evolved parental strains, following procedures described previously (100–102).

### Analytical procedures and kinetic parameters

The extracellular concentration of formate, acetate, and methanol was measured in culture supernatants. These samples, filtered through a 0.22-μm membrane, were analyzed by high-performance liquid chromatography in an UltiMate 3000 Basic Automated System (ThermoFisher Scientific Co.) equipped with an Aminex HPX-87P column (Bio-Rad) and a refractive index detector Shodex RI-101 (Showa Denko America Inc., New York, USA). Specific acetate (*q*_A_), formate (*q*_F_), and methanol (*q*_M_) consumption rates were determined according to the following equation:


$$q_{S}=\;\frac{1}{X}\frac{\Delta S}{\Delta t},$$

where *q*_S_ is the specific substrate consumption rate (mmol g_CDW_^−1^ h^−1^), *X* is the average biomass concentration between two sampling time points (g_CDW_ L^−1^), Δ*S* is the difference in substrate concentration between two sampling time points (mM), and Δ*t* refers to the time between two sampling points (h). The *q*_A_, *q*_F_, and *q*_M_ values were estimated by dividing μ by the slope of a linear regression in a plot of the biomass concentration over glucose concentration. CDW values were obtained from OD_600_ measurements by applying a strain-specific correlation factor, estimated by weighing the lyophilized cell pellet of a 50 mL culture harvested during the exponential phase.

### ^13^C-isotopic labeling of proteinogenic amino acids

An overnight LB culture grown at 30°C provided the inoculum for a preculture in MSM supplemented with the carbon sources and additives listed in the main text, under a headspace containing 10% (vol/vol) CO_2_. The cells from these precultures were washed with MSM without a carbon source and transferred to the appropriate medium, replacing the non-labeled carbon source with sodium ^13^C formate (Sigma-Aldrich Co.), sodium 1,2-^13^C-acetate (Cambridge Isotope Laboratories Inc., Andover, MA, USA), and/or ^13^CO_2_ (Cambridge Isotope Laboratories Inc.) as required for each experiment. Labeling experiments with formate and acetate were passaged twice in 100 mL Erlenmeyer flasks containing 10-mL of the corresponding medium to ensure isotopic steady state. These cultures were inoculated at an initial OD_600_ of 0.01. Cultures with ^13^C-formate as the sole carbon source were handled similarly but in 3 mL glass tubes. Experiments involving ^13^CO_2_ were conducted in airtight 6 L desiccators (Lab Companion, Daejeon, Korea) holding 3 mL tube cultures; the headspace was flushed with 10% (vol/vol) ^13^CO_2_ and 90% (vol/vol) air, then placed on a shaker platform at 200 rpm. When the stationary phase was reached, an amount of culture equivalent to 1 mL at OD_600_ of 0.5 was collected, washed once in Milli-Q water, and stored at –20°C. Proteinogenic amino acids were then extracted for biomass pellets, processed, and measured by GC-MS as previously described (103). Only ion fragments containing the complete carbon backbone of the corresponding amino acids—i.e., alanine^260^, glycine^246^, serine^390^, aspartate^418^, and glutamate^432^—were used for isotopologue analysis (103). Raw chromatographic data were integrated using SmartPeak (104), and the resulting mass-isotopomer distributions were corrected for the natural isotope content of all derivatization reagents (105).

### Genome-scale modeling simulations, flux balance analysis, STRING-based network analysis, and orthology studies

Flux balance analysis was used to estimate the optimal intracellular flux distribution of *P. putida* growing on formate or methanol through the rGlyP. Simulations relied on the most recent genome-scale metabolic reconstruction, *i*JN1463 (106–108), implemented in COBRApy (109). The native steps catalyzed by AceA, SerA, and LtaE were removed from the model, whereas the heterologous FtfL, Fch, and MtdA reactions were introduced as previously detailed (89). The flux distribution was optimized for biomass formation as the objective function, using the experimentally determined *q*_F_ and *q*_M_ values as constraints. STRING-based network and orthology analyses were carried out according to the approaches described by Martino et al. (110) and Pardo et al. (111), respectively.

### Statistical analysis

Data analysis was performed using MS Excel and Prism 9.0.2 (GraphPad Software Inc., San Diego, CA, USA). Reported values are indicated as averages ± standard deviation of replicates as specified in the legend of the corresponding figures. When relevant, the level of significance of differences when comparing results was evaluated by applying the Student’s *t* test (Prism 9.0.2) with α < 0.05.

## Data Availability

Data will be made available on request. All the raw genome sequencing data generated in this study are publicly available at the NCBI database with accession number PRJNA1281011.

## SUPPLEMENTAL MATERIAL

Supplemental Material mbio.01976-25-s0001.pdf Supplemental figures and tables

ASM does not own the copyrights to Supplemental Material that may be linked to, or accessed through, an article. The authors have granted ASM a non-exclusive, world-wide license to publish the Supplemental Material files. Please contact the corresponding author directly for reuse.
