# Supplementary material for: Synthetic C1 metabolism in Pseudomonas putida enables strict formatotrophy and methylotrophy via the reductive glycine pathway
Source: mBio. 2025 Aug 18;16(9):e01976-25. doi: 10.1128/mbio.01976-25 (PMC12421819; doi:10.1128/mbio.01976-25)
Supplement: Supplemental Material — Supplemental figures and tables [file mbio.01976-25-s0001.pdf]

SUPPLEMENTAL MATERIAL

Synthetic C_1 metabolism in *Pseudomonas putida* enables strict formatotrophy and methylotrophy *via* the reductive glycine pathway

by

Justine Turlin [1], Maria V. G. Alván-Vargas [2], Òscar Puiggené [1], Stefano Donati [1], Sebastian Wenk [2,3], and Pablo I. Nikel [1*]

1 The Novo Nordisk Foundation Center for Biosustainability, Technical University of Denmark, Kongens Lyngby, Denmark
2 Max Planck Institute of Molecular Plant Physiology, Potsdam, Germany
3 Faculty of Science and Engineering, University of Groningen, Netherlands

Running title: Synthetic C_1-trophy in *P. putida*

* Correspondence to:	*Pablo I. Nikel* (pabnik@biosustain.dtu.dk)<br>Tel: +45 93 5119 18<br>The Novo Nordisk Foundation Center for Biosustainability<br>Technical University of Denmark<br>2800 Kongens Lyngby, Denmark

Fig. S1. Reverse engineering of adaptive mutations explains enhanced growth on formate, acetate, and CO_2.

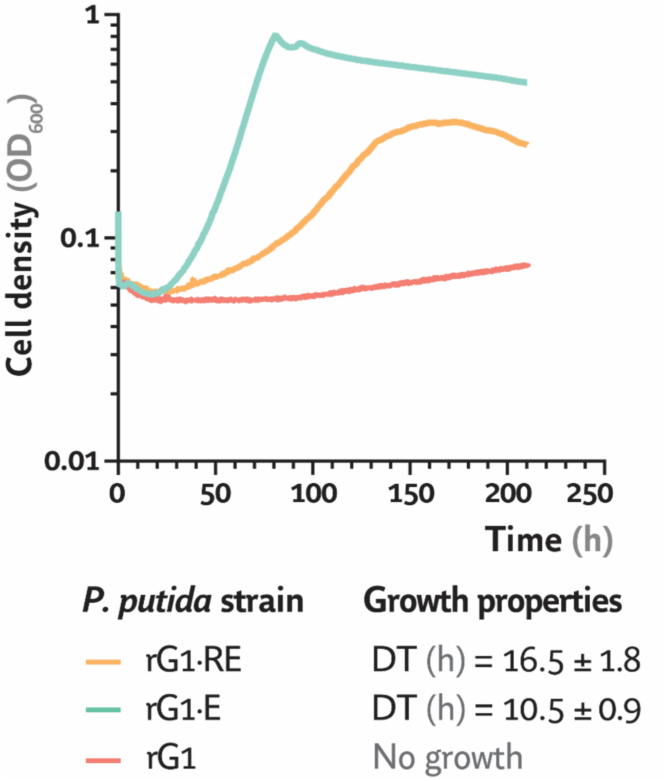

Growth profile of *P. putida* rG1, rG1·E, and rG1·RE. Strains were cultivated in microtiter plates in MSM supplemented with 60 mM formate, 20 mM acetate, and 10% (v/v) CO_2 in the headspace. The average cell density (estimated as the optical density at 600 nm, OD_{600}) of three independent replicates is represented. *DT*, doubling time.

Fig. S2. Screening of clones upon adaptive laboratory evolution of a *P. putida* strain engineered for growth on formate and CO_2.

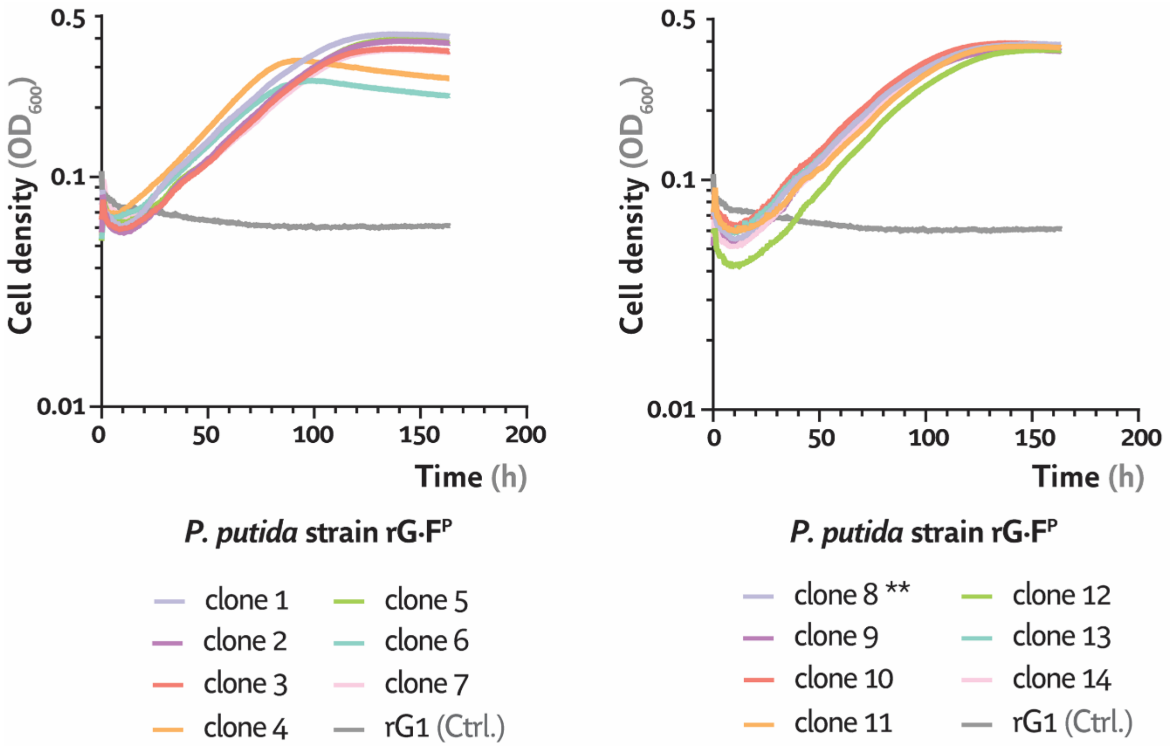

Growth patterns of 14 clones isolated from the evolved population of rG·F^P after >1 month of ALE. rG·F^P clone 8 (**) was chosen to continue further experiments. Strains were grown in microtiter plates in MSM supplemented with 80 mM formate and 10% (v/v) CO_2 in the headspace. The average cell density (estimated as the OD_{600}) of three independent replicates is represented. Strain rG1 was included in these cultures as a control (Ctrl.).

Table S1. Oligonucleotides used in this study.

Name	DNA sequence[a] (5'→3')	Use
*folM**-U-US-F	AGATCCUGGCGTTTTTCCCAGTTTCG	Reverse engineering
*folM**-U-US-R	ATGGTGCGUAGTCCGTCATAACGATCGT	
*folM**-U-DS-F	ACGCACCAUCATCCACAACGCCTCGCTAT	
*folM**-U-DS-R	AGGTCGACUCTGTTGCTGTGGGGTCATGGT	
*sdhA**-U-US-F	AGATCCUGAGCGTTACGCGCCGAACGCCAAA	Reverse engineering
*sdhA**-U-US-R	AGGGCAACAUCGACGTCGGTGTCGCTGGCG	
*sdhA**-U-DS-F	ATGTTGCCCUCCGCCTGAACAAGCTCAACGA	
*sdhA**-U-DS-R	AGGTCGACUGCCGAGTCGGTGTCCGGGTTGT	
P4*M1-F	ATCCAGCTCTAGAATAGATATCCTATAAAATATTCAG	Reverse engineering
P4*M1-R	ATTATACAGAAAAATTTTCCTGATGTCAATTAATT	
pS221-U-F	AATGCGUAAAGGTGAAGAACTGTTC	Construction of a transcriptional reporter plasmid
pS221-U-R	AAAGGCAUCAAATAAAACGAAAGGCT	
BCD10-U-F	ATGCCTTUAATTAAGCCCATTGACAAGGCTCTC	
BCD10-U-R	ACGCATUAGAAACGATCCTCCGCAT	
P$_4$*GFP-F	ATCCAGCTCTAGAATAGATATCCTATAAAATATTCAG	Transcriptional reporter assembly
P$_4$*GFP-R	ATTATACAGAAAAATTTTCCTGATGTCAATTAATT	
pS221-P4*M1-U-F	ATAAACUAGTCTTGGACTCCTGTTG	Transcriptional reporter assembly
pS221-P4*M1-U-R	ATAAAAGGUCCTCCTTCTGAATATTTTATAG	
GFP-U-F	ACCTTTTAUGCGTAAAGGTGAAGAACT	
GFP-U-R	AGTTTAUTTGTAGAGTTCATCCATGC	
BCD10*-F	GGCCAGGTATAATTGCACGAGAGCCCAAGTTCACTTAAAA	Transcriptional reporter
BCD10*-R	GCGAGAGCCTTGTCAATGGGCGGGGAA	
EMG-F	ATCTAGAGUCGACCTGCAGGCATGCAA	Construction of pEGM derivatives
EMG-R	ATATGTUTTTCCTCCTGGGAATTCGC	
FDH-F	AACATAUGCATCATCACCATCACCA	
FDH-R	ACTCTAGAUTAAACAGCTTTTTTGAATTTAGC	
pBAMD-U-US-F	TCTAGAGTCGACCTGCAGGCATGCAAGCTT	Construction of mini-Tn*5* libraries for *fdh*
pBAMD-U-US-R	GGATCCCCGGGTACCGAGCTCGAATTCGCG	
FDH-U-DS-F	ATGCATCATCACCATCACCACG	
FDH-U-DS-R	GCTAAATTCAAAAAAGCTGTTTAATCTAGAGTCGACCTGCAG GCATGCAAGCTT	
P-BCD-U-F	CGCGAATTCGAGCTCGGTACCCGGGGATCCCGGAGTTGACAA CACTCGAAAAG	
P-BCD-U-R	ATCATGCAATGGAGGCTTTCTAATGCATCATCACCATCACCA CGCTAAAGTT	
pBAMD_^{Cn}*mdh*_F	ATGTAATCUAGAGTCGACCTGCAGGCA	Construction of mini-Tn*5* libraries for ^{Cn}*mdh*
pBAMD_^{Cn}*mdh*_R	AGGTGCGTGUGGTGATGGTGATGATGCATT	
^{Cn}*mdh*_pBAMD_F	ACACGCACCUCAACATTGCGAACCGTGTCGA	
^{Cn}*mdh*_pBAMD_R	AGATTACAUGGCCGCTGCGAAGATAGC	
pGNW-USER-F	AGTCGACCUGCAGGCATGCAAGCTTCT	Amplification of vector pGNW2
pGNW-USER-R	AGGATCUAGAGGATCCCCGGGTACCG	

[a] USER plasmids contain U residues to facilitate assembly (1).

Table S2. Codon-optimized gene sequences used for engineering *P. putida*.

Enzyme	UniProt entry and reference	DNA sequence (5'→3')
Formate-tetrahydrofolate ligase from *Methylobacterium extorquens* AM1	Q83WS0; Turlin *et al.* (2)	ATGCCCTCAGATATCGAGATCGCCCGCGCGGCGACCCTGAAGCCGATCG<br>CCCAGGTCGCCGAAAAGCTCGGCATCCCGGACGAGGCGCTTCACAACTA<br>CGGCAAGCACATCGCCAAGATCGACCACGACTTCATCGCCTCGCTCGAG<br>GGTAAGCCCGAGGGCAAGCTGGTGCTCGTCACCGCGATCTCGCCGACGC<br>CCGCGGGCGAGGGCAAGACCACCACGACCGTGGGTCTCGGCGACGCACT<br>CAACCGGATCGGCAAGCGGGCGGTGATGTGCCTGCGCGAGCCCTCGCTC<br>GGCCCCTGCTTCGGCATGAAGGGCGGCGCGGCCGGTGGCGGCAAGGCCC<br>AGGTCGTGCCGATGGAGCAGATCAACCTGCACTTCACCGGGGACTTCCA<br>CGCCATCACCTCGGCGCACTCGCTCGCCGCCGCGCTGATCGACAACCAC<br>ATCTACTGGGCCAACGAGCTCAACATCGACGTGCGCCGCATCCACTGGC<br>GCCGCGTGGTCGACATGAACGACCGGGCGCTGCGCGCGATCAACCAGTC<br>GCTCGGCGGCGTCGCCAACGGCTTTCCGCGTGAGGACGGGTTCGACATC<br>ACCGTCGCCTCCGAGGTGATGGCGGTGTTCTGCCTCGCCAAGAATCTGG<br>CCGACCTCGAGGAGCGGCTCGGCCGCATCGTCATCGCCGAGACCCGCGA<br>CCGCAAGCCGGTGACGCTGGCCGACGTGAAGGCGACCGGCGCGATGACC<br>GTTCTCCTCAAGGATGCGCTGCAGCCGAACCTCGTGCAGACGCTGGAGG<br>GCAACCCGGCCCTGATCCATGGCGGCCCGTTCGCCAACATCGCCCACGG<br>CTGCAACTCGGTGATCGCCACCCGTACCGGCCTGCGGCTGGCCGACTAC<br>ACCGTCACCGAGGCCGGCTTCGGCGCGGATCTCGGCGCGGAGAAGTTCA<br>TCGACATCAAGTGCCGCCAGACCGGCCTCAAGCCCTCGGCGGTGGTGAT<br>CGTCGCCACGATCCGCGCCCTCAAGATGCATGGCGGCGTCAACAAGAAG<br>GATCTCCAGGCTGAGAACCTCGACGCGCTGGAGAAGGGTTTCGCCAACC<br>TCGAGCGCCACGTGAACAACGTGCGGAGCTTCGGCCTGCCGGTGGTGGT<br>GGGCGTGAACCACTTCTTCCAGGACACCGACGCCGAGCATGCCCGGTTG<br>AAGGAGCTCTGCCGCGACCGTCTTCAGGTCGAGGCGATCACCTGCAAGC<br>ACTGGGCGGAGGGCGGCGCGGGCGCCGAGGCTCTGGCGCAGGCCGTGGT<br>GAAGCTCGCCGAGGGCGAGCAGAAGCCGCTGACCTTCGCCTACGAAACT<br>GAGACGAAGATCACCGACAAGATCAAGGCGATCGCGACCAAGCTCTACG<br>GTGCGGCCGATATCCAGATCGAGTCGAAGGCCGCCACCAAGCTCGCCGG<br>CTTCGAGAAGGATGGCTACGGCGGATTGCCCGTCTGCATGGCCAAGACG<br>CAGTACTCGTTCTCGACCGACCCGACCCTGATGGGCGCGCCCTCGGGCC<br>ACCTCGTCTCGGTGCGCGACGTGCGCCTCTCGGCGGGCGCCGGCTTCGT<br>CGTGGTGATCTGCGGTGAGATCATGACCATGCCGGGCCTGCCCAAGGTG<br>CCGGCGGCGGACACCATCCGCCTCGACGCCAACGGTCAGATCGACGGGC<br>TGTTCTAG
5,10-Methenyltetrahydrofolate cyclohydrolase from *Methylobacterium extorquens* AM1	Q49135; Turlin *et al.* (2)	ATGGCCGGCAACGAGACGATCGAAACATTCCTCGATGGCCTGGCGAGCT<br>CGGCCCCGACCCCCGGCGGCGGCGGTGCCGCCGCGATCTCCGGCGCCAT<br>GGGCGCGGCGCTGGTCTCGATGGTGTGTAACCTCACCATCGGCAAGAAG<br>AAGTATGTCGAGGTCGAGGCCGACCTGAAGCAGGTGCTGGAGAAGTCGG<br>AAGGCCTGCGCCGCACGCTCACCGGCATGATCGCCGACGACGTCGAGGC<br>TTTCGACGCGGTGATGGGCGCCTACGGGCTGCCGAAAAACACCGACGAG<br>GAGAAGGCTGCCCGCGCCGCCAAGATTCAGGAGGCGCTCAAGACCGCGA<br>CCGACGTGCCGCTCGCCTGCTGCCGCGTCTGCCGCGAGGTGATCGATCT<br>GGCCGAGATCGTCGCCGAGAAGGGCAATCTCAACGTCATCTCGGATGCC<br>GGCGTCGCCGTGCTCTCGGCCTATGCCGGTCTGCGCTCGGCGGCCCTCA<br>ACGTCTACGTCAACGCCAAGGGCCTCGACGACCGCGCCTTCGCCGAGGA<br>GCGGCTGAAGGAGCTGGAAGGCCTGCTGGCCGAGGCGGGCGCGCTCAAC<br>GAGCGGATCTACGAGACGGTGAAGTCCAAGGTAAACTGA

5,10-Methylenetetrahydrofolate dehydrogenase from *Methylobacterium extorquens* AM1	P55818;<br>Turlin *et al.* (2)	ATGTCCAAGAAGCTGCTCTTCCAGTTCGACACCGATGCCACGCCGAGCG<br>TCTTCGACGTCGTCGTCGGCTACGACGGCGGTGCCGACCACATCACCGG<br>CTACGGCAACGTCACGCCCGACAACGTCGGCGCCTATGTCGACGGCACG<br>ATCTACACCCGCGGCGGCAAGGAGAAGCAGTCGACGGCGATCTTCGTCG<br>GCGGCGGCGACATGGCGGCCGGCGAGCGGGTGTTCGAGGCGGTGAAGAA<br>GCGCTTCTTCGGCCCGTTCCGCGTGTCCTGCATGCTGGATTCGAACGGC<br>TCCAACACGACCGCCGCGGCGGGTGTGGCGCTCGTCGTCAAGGCGGCGG<br>GCGGCTCGGTCAAGGGCAAGAAGGCCGTCGTGCTCGCGGGCACCGGCCC<br>GGTCGGCATGCGCTCGGCGGCGCTGCTCGCCGGCGAGGGCGCCGAGGTC<br>GTGCTGTGCGGGCGCAAGCTCGACAAGGCGCAGGCCGCGGCCGATTCCG<br>TGAACAAGCGCTTCAAGGTGAACGTCACCGCGGCCGAAACCGCGGACGA<br>CGCTTCGCGCGCCGAGGCCGTGAAGGGCGCCCATTTCGTCTTCACCGCC<br>GGTGCGATCGGCCTTGAACTGCTGCCGCAGGCAGCCTGGCAGAACGAGA<br>GTTCGATCGAGATCGTGGCCGACTACAACGCCCAGCCGCCGCTCGGCAT<br>CGGCGGGATCGATGCGACCGACAAAGGCAAGGAATACGGCGGAAAGCGC<br>GCCTTCGGTGCGCTCGGCATCGGCGGCTTGAAGCTCAAGCTGCACCGCG<br>CCTGCATCGCCAAGCTGTTCGAGTCGAGCGAAGGCGTCTTCGACGCCGA<br>GGAGATCTACAAGCTGGCCAAGGAAATGGCCTGA
Formate dehydrogenase from *Pseudomonas* sp. strain 101	P33160;<br>Lazmin *et al.* (3)	ATGCATCATCACCATCACCACGCTAAAGTTCTGTGCGTTCTGTACGACG<br>ACCCGGTTGACGGTTACCCGAAAACCTACGCTCGTGACGACCTGCCGAA<br>AATCGACCACTACCCGGGTGGTCAGACCCTGCCGACCCCGAAAGCTATC<br>GACTTCACCCCGGGTCAGCTGCTGGGTTCTGTTTCTGGTGAACTGGGTC<br>TGCGTAAATACCTGGAATCTAACGGTCACACCCTGGTTGTTACCTCTGA<br>CAAAGACGGTCCGGACTCTGTTTTCGAACGTGAACTGGTTGACGCTGAC<br>GTTGTTATCTCTCAGCCGTTCTGGCCGGCTTACCTGACCCCGGAACGTA<br>TCGCTAAAGCTAAAAACCTGAAACTGGCTCTGACCGCTGGTATCGGTTC<br>TGACCACGTTGACCTGCAATCTGCTATCGACCGTAACGTTACCGTTGCT<br>GAAGTTACCTACTGCAACTCTATCTCTGTTGCTGAACACGTTGTTATGA<br>TGATCCTGTCTCTGGTTCGTAACTACCTGCCGTCTCACGAATGGGCTCG<br>TAAAGGTGGTTGGAACATAGCTGACTGCGTAAGCCACGCTTACGACCTG<br>GAAGCTATGCACGTTGGTACCGTTGCTGCTGGTCGTATCGGTCTGGCTG<br>TTCTGCGTCGTCTGGCTCCGTTCGACGTTCACCTGCACTACACCGACCG<br>TCACCGTCTGCCGGAATCTGTTGAAAAAGAACTGAACCTGACCTGGCAC<br>GCTACCCGTGAAGACATGTACCCGGTTTGCGACGTTGTTACCCTGAACT<br>GCCCGCTGCACCCGGAAACCGAACACATGATCAACGACGAAACCCTGAA<br>ACTGTTCAAACGTGGTGCTTACATCGTTAACACCGCTCGTGGTAAACTG<br>TGCGACCGTGACGCTGTTGCTCGTGCTCTGGAATCTGGTCGTCTGGCTG<br>GTTATGCGGGTGACGTGTGGTTCCCCCAGCCGGCTCCGAAAGACCACCC<br>GTGGCGTACCATGCCGTACAACGGTATGACCCCGCACATCTCTGGTACC<br>ACCCTGACCGCTCAGGCTCGTTACGCTGCTGGTACCCGTGAAATCCTGG<br>AATGCTTCTTCGAAGGTCGTCCGATCCGTGACGAATACCTGATCGTTCA<br>GGGTGGTGCTCTGGCTGGTACCGGTGCTCACTCTTACTCTAAAGGTAAC<br>GCTACCGGTGGTTCTGAAGAAGCTGCTAAATTCAAAAAAGCTGTTTAA

Methanol dehydrogenase CT4-1. engineered from *C. necator* N-1	F8GNE5; codon-optimized sequence reported by Wu *et al.* (4), contains the A26V, A31V, and A169V mutations of *mdh2*	ATGCATCATCACCATCACCACACGCACCTCAACATTGCGAACCGTGTCG<br>ACTCCTTCTTCATCCCATGTGTGACCCTGTTCGGGCCAGGTTGTGTGCG<br>TGAAACGGGGGTGCGCGCTCGGTCCCTCGGTGCCCGGAAGGCACTCATC<br>GTGACCGACGCGGGCTTGCACAAGATGGGCCTCAGCGAGGTGGTGGCTG<br>GCCACATCCGTGAAGCTGGCCTGCAAGCAGTGATCTTCCCCGGGGCAGA<br>GCCTAACCCGACCGATGTGAATGTGCATGACGGGGTCAAACTCTTCGAA<br>CGCGAAGAGTGCGACTTCATCGTGTCGCTCGGTGGCGGCTCCAGCCACG<br>ACTGTGCGAAAGGGATTGGGCTCGTCACCGCGGGTGGCGGCCACATTCG<br>GGATTACGAAGGGATCGACAAAAGCACGGTGCCTATGACCCCCTTGATC<br>TCCATTAATACGACCGCAGGTACCGCAGCAGAGATGACGCGCTTCTGTA<br>TTATCACGAATTCGTCGAACCATGTCAAAATGGTGATTGTCGACTGGCG<br>TTGTACCCCCTTGATCGCGATTGATGATCCGTCGCTGATGGTGGCTATG<br>CCTCCGGCTTTGACCGCTGCCACGGGGATGGACGCACTGACCCATGCGA<br>TCGAGGCCTATGTGTCGACGGCTGCAACGCCAATCACGGATGCTTGTGC<br>CGAAAAAGCAATTGTGTTGATTGCCGAATGGCTGCCTAAGGCGGTGGCA<br>AACGGGGATAGCATGGAAGCACGTGCTGCTATGTGCTATGCCCAGTACC<br>TCGCGGGTATGGCTTTTAATAATGCGTCGTTGGGGTACGTCCACGCGAT<br>GGCTCATCAACTGGGTGGCTTTTACAACTTGCCCCACGGTGTGTGTAAC<br>GCCATCCTCCTCCCACATGTGTCCGAGTTTAATTTGATTGCCGCCCCCG<br>AACGGTACGCACGTATTGCGGAGCTCTTGGGTGAGAATATCGGGGGCCT<br>CTCGGCCCATGATGCAGCAAAGGCCGCAGTGTCGGCGATCCGTACCTTG<br>TCCACGTCCATCGGGATTCCCGCAGGTCTGGCCGGGCTGGGCGTCAAAG<br>CCGACGACCATGAGGTCATGGCCTCCAACGCTCAAAAAGATGCGTGTAT<br>GCTCACGAACCCGCGCAAGGCAACGCTGGCCCAAGTCATGGCTATCTTC<br>GCAGCGGCCATGTAA

Table S3. Key mutations associated with mixotrophic, formatotrophic, and methylotrophic growth in engineered *P. putida* evolved under different ALE regimes.[a]

Selection regime	Locus	Mutation type[b]	Potential effect(s)[c] and references
Mixotrophic	P_4 promoter	Deletion (partial)	Increase in promoter strength
	PP_4632 (folM, bifunctional dihydrofolate reductase/ dihydromonapterin reductase	SNP, substitution	Function unclear; FolM catalyzes the reduction of 7,8-dihydrofolate to THF, essential for the initial formate activation step
	PP_4191 (*sdhA*, succinate dehydrogenase, flavoprotein subunit)	SNP, substitution	Function unclear; this mutation affects a key step (succinate dehydrogenase) in the TCA cycle
	Intergenic region, BCD10 translational coupler	SNP, substitution	Increase in translational coupling efficiency (5) (affecting *gcvH-I*)
Formatotrophic	*PP_2683* (two-component system sensor histidine kinase/response regulator)	Transposon (*PP_4459*) insertion	Function unclear; PP_2683 is part of a two-component system that regulates the *ped* cluster (6-8) and this insertion may silence the expression of *ped* genes, responsive to methanol and other alcohols (9)
	PP_1261 (*ghrB*, 2-ketoaldonate reductase, hydroxypyruvate, glyoxylate reductase) and *PP_1262* (LysR family transcriptional regulator)	Deletion (total)	Function unclear; GhrB catalyzes the NADPH-dependent reduction of glyoxylate to glycolate (10) and eliminating this activity could help carbon conservation and redox balance
	PP_0298 (*gbdR*, AraC family transcriptional regulator)	Deletion (partial)	Function unclear; AraC-type regulator involved in nitrogen metabolism, probably due to higher levels of glycine or serine
	P_{EM7} promoter	Deletion (partial)	Increase in promoter strength (affecting *pntAA*)
	PP_4121 (*nuoC*, NADH-quinone oxidoreductase subunit C/D)	Deletion, introduces a frameshift that creates a premature *STOP* codon	Function unclear; this mutation may tune down the levels and activity of the Nuo respiratory complex
	P_{EM7} promoter	Deletion (partial)	Function unclear; potentially altered promoter strength (affecting *gcvTHP-II*)
	PP_5194 (*gcvT-II*, aminomethyltransferase)	SNP, creates a premature *STOP* codon	Function unclear; may tune GcvT-II levels
	Intergenic region between *PP_0987* (*tdcG-II*) and *PP_0988* (*gcvP-I*)	Single-nucleotide insertion	Function unclear; this mutation may affect transcription levels of *tdcG-II* or translational stability of *gcvP-I*
	PP_4156 (LysR family transcriptional regulator)	Mini-Tn*5* insertion (engineered)	Function unclear; this insertion disrupts the regulator without impairing formatotrophy; the inserted expression cassette drives chromosomal *fdh* expression

Methylotrophic	*PP_5029* (*N*-formyl-L-glutamate deformylase)	Mini-Tn*5* insertion (engineered)	Function unclear; site of transposon insertion expressing optimized ^{Cn}*mdh* methanol dehydrogenase cassette

[a] The complete list of mutations is available in the genome sequences deposited in GenBank as part of this study.

[b] Mutations labeled as "engineered" were intentionally introduced during strain construction. *SNP*, single nucleotide polymorphism.

[c] The potential effects of mutations are indicated as "Function unclear" when they have not been experimentally validated.

References

1. Nour-Eldin HH, Geu-Flores F, Halkier BA. 2010. *USER* cloning and *USER* fusion: The ideal cloning techniques for small and big laboratories. Methods Mol Biol 643:185–200. https://doi.org/10.1007/978-1-60761-723-5_13.
2. Turlin J, Dronsella B, De Maria A, Lindner SN, Nikel PI. 2022. Integrated rational and evolutionary engineering of genome-reduced *Pseudomonas putida* strains promotes synthetic formate assimilation. Metab Eng 74:191–205. https://doi.org/10.1016/j.ymben.2022.10.008.
3. Lamzin VS, Aleshin AE, Strokopytov BV, Yukhnevich MG, Popov VO, Harutyunyan EH, Wilson KS. 1992. Crystal structure of NAD-dependent formate dehydrogenase. Eur J Biochem 206:441–452. https://doi.org/10.1111/j.1432-1033.1992.tb16945.x.
4. Wu TY, Chen CT, Liu JT, Bogorad IW, Damoiseaux R, Liao JC. 2016. Characterization and evolution of an activator-independent methanol dehydrogenase from *Cupriavidus necator* N-1. Appl Microbiol Biotechnol 100:4969–4983. https://doi.org/10.1007/s00253-016-7320-3.
5. Zobel S, Benedetti I, Eisenbach L, de Lorenzo V, Wierckx NJP, Blank LM. 2015. Tn7-Based device for calibrated heterologous gene expression in *Pseudomonas putida*. ACS Synth Biol 4:1341–1351. https://doi.org/10.1021/acssynbio.5b00058.
6. Wehrmann M, Berthelot C, Billard P, Klebensberger J. 2018. The PedS2/PedR2 two-component system is crucial for the rare Earth element switch in *Pseudomonas putida* KT2440. mSphere 3:e00376–18. https://doi.org/10.1128/mSphere.00376-18.
7. Wehrmann M, Berthelot C, Billard P, Klebensberger J. 2019. Rare Earth element (REE)-dependent growth of *Pseudomonas putida* KT2440 relies on the ABC-transporter PedA1A2BC and is influenced by iron availability. Front Microbiol 10:2494. https://doi.org/10.3389/fmicb.2019.02494.
8. Wehrmann M, Billard P, Martin-Meriadec A, Zegeye A, Klebensberger J. 2017. Functional role of lanthanides in enzymatic activity and transcriptional regulation of pyrroloquinoline quinone-dependent alcohol dehydrogenases in *Pseudomonas putida* KT2440. mBio 8:00570–17. https://doi.org/10.1128/mBio.00570-17.
9. Turlin J, Puiggené Ò, Donati S, Wirth NT, Nikel PI. 2023. Core and auxiliary functions of one-carbon metabolism in *Pseudomonas putida* exposed by a systems-level analysis of transcriptional and physiological responses. mSystems 8:e00004–23. https://doi.org/10.1128/msystems.00004-23.
10. Franden MA, Jayakody LN, Li WJ, Wagner NJ, Cleveland NS, Michener WE, Hauer B, Blank LM, Wierckx N, Klebensberger J, Beckham GT. 2018. Engineering *Pseudomonas putida* KT2440 for efficient ethylene glycol utilization. Metab Eng 48:197–207. https://doi.org/10.1016/j.ymben.2018.06.003.
